# GIS-NaP1 zeolite microspheres as potential water adsorption material: Influence of initial silica concentration on adsorptive and physical/topological properties

**DOI:** 10.1038/srep22734

**Published:** 2016-03-11

**Authors:** Pankaj Sharma, Ju-Sub Song, Moon Hee Han, Churl-Hee Cho

**Affiliations:** 1 Graduate School of Energy Science and Technology, Chungnam National University, 99 Daehak-ro, Yuseong-gu, Daejeon 305-764, Republic of Korea

## Abstract

GIS-NaP1 zeolite samples were synthesized using seven different Si/Al ratios (5–11) of the hydrothermal reaction mixtures having chemical composition Al_2_O_3_:*x*SiO_2_:14Na_2_O:840H_2_O to study the impact of Si/Al molar ratio on the water vapour adsorption potential, phase purity, morphology and crystal size of as-synthesized GIS-NaP1 zeolite crystals. The X-ray diffraction (XRD) observations reveal that Si/Al ratio does not affect the phase purity of GIS-NaP1 zeolite samples as high purity GIS-NaP1 zeolite crystals were obtained from all Si/Al ratios. Contrary, Si/Al ratios have remarkable effect on the morphology, crystal size and porosity of GIS-NaP1 zeolite microspheres. Transmission electron microscopy (TEM) evaluations of individual GIS-NaP1 zeolite microsphere demonstrate the characteristic changes in the packaging/arrangement, shape and size of primary nano crystallites. Textural characterisation using water vapour adsorption/desorption, and nitrogen adsorption/desorption data of as-synthesized GIS-NaP1 zeolite predicts the existence of mix-pores i.e., microporous as well as mesoporous character. High water storage capacity 1727.5 cm^3^ g^−1^ (138.9 wt.%) has been found for as-synthesized GIS-NaP1 zeolite microsphere samples during water vapour adsorption studies. Further, the total water adsorption capacity values for P6 (1299.4 mg g^−1^) and P7 (1388.8 mg g^−1^) samples reveal that these two particular samples can absorb even more water than their own weights.

In past couple of decades, significant research has been made to explore the applicability of micro- and mesoporous adsorbent materials in open and closed system heat transformation applications^1^,^2^,^3^,^4^,^5^. The adsorption of water by porous solids is important for many applications those necessitate capture and release of water such as electric dehumidifier, adsorption heat pumps (AHPs), alcohol/organic solvent dehydration etc. Recent research on AHPs have primarily focused on the development of more environment-friendly systems that can provide heating and cooling effects by utilizing low-grade thermal energy sources such as solar and geothermal energies or waste heat from a variety of industrial processes. One of the most promising technologies in this context is based on the evaporation and consecutive adsorption of coolant liquids, preferably water, under specific conditions^5^,^6^. Considering these potential environmental and ecological problems, an issue that come under increasing scrutiny in recent years is the interaction between water vapours and porous adsorbents. Moreover, to improve the quality and safe storage of processed foods and moisture sensitive materials, the need for moisture removal technology is also becoming important^7^,^8^,^9^. In daily human life relative humidity is also an important factor as it effects the health. Highly humid environment makes house dust mites, provide favourable environment for fungi and harmful bacteria to grow, destruct the hot-humidity balance of human body etc.^10^. Further, water is also present in several gaseous industrial streams such as, methane reforming hydrogen production, natural gas etc.^11^. Water adsorption behaviour of porous materials play vital role in manufacturing and designing of advance devices. Therefore, the demand for controlling the humidity and development of high efficiency sorbent technology and AHPs enhances the great interest in new porous materials especially nano/microporous^4^. The adsorption based cooling process is a fundamental and a long lasting technique, dating back to Faraday (1848)^12^.

The worldwide energy consumption is kept on growing and raising concerns about future energy supplies and resources. Heating and cooling systems are widely used in industrial and building sector, and the share of the energy for these two purposes in total energy consumption increases a lot^13^. Water heating is the fourth largest energy user in the commercial building sector, after air conditioning, heating and light^14^. In general, building heating and cooling accounts nearly 50% of the total energy consumption^15^. Most water heaters are equipped with conventional heaters generating heat by consuming fossil fuels, electricity and gas. But these conventional/mechanical heat pumps and refrigeration system are the key factors in global warming and the depletion of ozone layer. With increasing concerns over ozone depletion and the global warming potential of chlorofluorocarbons and Hydrofluorocarbons, augmented energy demand and the resulting CO_2_ emissions, the interest in energy-efficient systems and especially, new cooling and heating technologies that make use of environmentally friendly refrigerants has grown rapidly^5^. Thus, efficient use of energy for heating and cooling of building is a key issue towards a sustainable and secure energy supply in future^16^. A variety of porous materials (zeolites, metal organic frameworks, carbon based adsorbents, organic polymers) have been explored for all these applications but still it remains a challenge to find materials of high performance combining high water uptake, precise operational pressure range control, recyclability, stability etc.^2^,^17^,^18^,^19^,^20^,^21^,^22^,^23^.

Zeolite P (also termed as zeolite B) is the synthetic analogue of the gismondine-type (GIS-type) zeolites and has a two-dimensional pore system with two intersecting 8-membered oxygen ring channels of 0.31 × 0.44 nm and 0.26 × 0.49 nm in the [100] and [010] directions, respectively. Smaller micropore size of zeolite P (~2.9 Å) than that of MFI (~5.4 Å), FAU (~7.4 Å), LTA (~4.1 Å), LTL (~7.1 Å) and MOR (~6.7 Å) zeolite types makes it useful and valuable applicant for the water vapour adsorption and separation of small molecules. GIS-NaP1 zeolite type is also useful for the removal of radio tracer elements, heavy metals and organic matter from aqueous waste, seawater potassium extraction and formation of environmental friendly detergent^24^. Beside, GIS-NaP1 zeolite exhibits knobbed surface spherical morphology because spheres actually form the assembling of nano-size particles, and this imparts mesoporous character to the same^25^. Furthermore, hierarchical porous structure of self-assembled GIS-NaP1 zeolite microspheres containing micro-, meso- as well as macropores can significantly improve the diffusion of guest molecules, transport resistance, etc., compared with single-sized micropore materials which consequentially increase mass transport through the material and maintenance of a specific surface area on the level of fine pore systems. Moreover, in comparison to other zeolite types it is difficult to distinguish between different NaP phases and to understand their composition and structure. Although all zeolite P are characterised by the same framework topology GIS type zeolite, the formula, symmetry and structure of samples of different composition (Si/Al ratio, exchangeable cations etc.) were found to be difficult to establish. The main reason for this state of affairs is the high flexibility of the Si–O–Al linkage in aluminosilicate framework, which has been described being the most open tetrahedral framework type generated so far^26^. Although, crystalline porous MOFs materials are consider to be promising water adsorbent but those appears to be structurally unstable or having low water stability.

Considering the importance of water adsorption, water adsorption-desorption based applications, and structural properties of GIS-NaP1 zeolite an attempt was made in the present work to synthesize high purity, porous GIS-NaP1 zeolite microspheres with high water uptake capacity for their greater application in dehumidification, thermal driven adsorption chiller (TDCs) or AHPs, and delivery of drinking water in desert areas and arid regions. The importance of morphology and particle size is well established for the application of microporous solids to adsorption, separation and many other applications, therefore, effect of Si/Al ratio on the phase purity, morphology and crystal size of GIS-NaP1 zeolite microspheres has been investigated. As the prime focus of this study to develop novel water adsorption material, therefore, effect of Si/Al ratio on the water adsorption potential of as-synthesized GIS-NaP1 zeolite microspheres has also been studied.

## Results

### Phase purity and morphological evaluation of as-synthesized GIS-NaP1 zeolite

**Figure 1:**
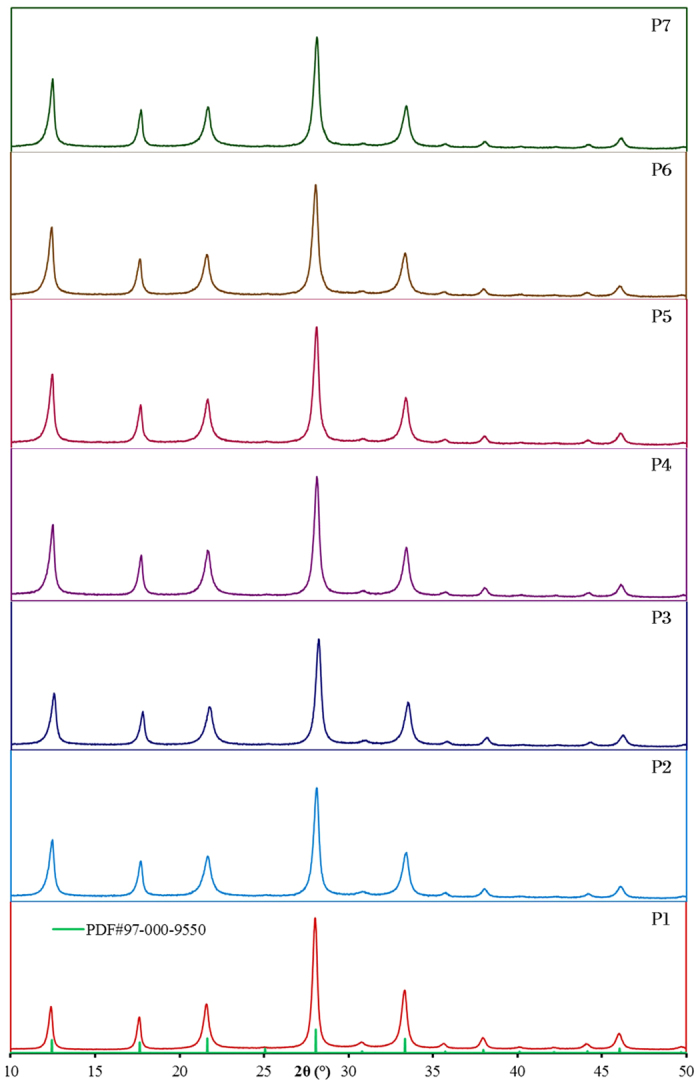
XRD diffraction patterns of as-synthesized GIS-NaP1 zeolite microspheres and JCPDS file no. PDF#97-000-9550 data showing high phase purity of each sample.

The XRD patterns of as-synthesized GIS-NaP1 zeolite microspheres obtained from different Si/Al ratio precursor reaction mixtures are shown in Fig. 1 and phase identification was made by comparing them with standard XRD data (JCPDS file no. PDF#97-000-9550). These XRD patterns reveal that all GIS-NaP1 zeolite samples have single phase material which indicates the high phase purity of these samples. The peak width of these XRD peaks and crystallite size derived from Scherrer formula indicate that each GIS-NaP1 zeolite microsphere is composed of nano crystallites. Furthermore, there is no change observed in the peak positions with increase in the Si/Al ratio except small variation in the peak intensities. These peak intensity variations are most probably due to the change in size of crystallites of which GIS-NaP1 zeolite microspheres were composed of. It indicates that even though we increased the Si/Al ratio from 5 to 11 in the reaction mixture but still we are able to synthesize highly crystalline, phase pure GIS-NaP1 zeolite crystals.

**Figure 2:**
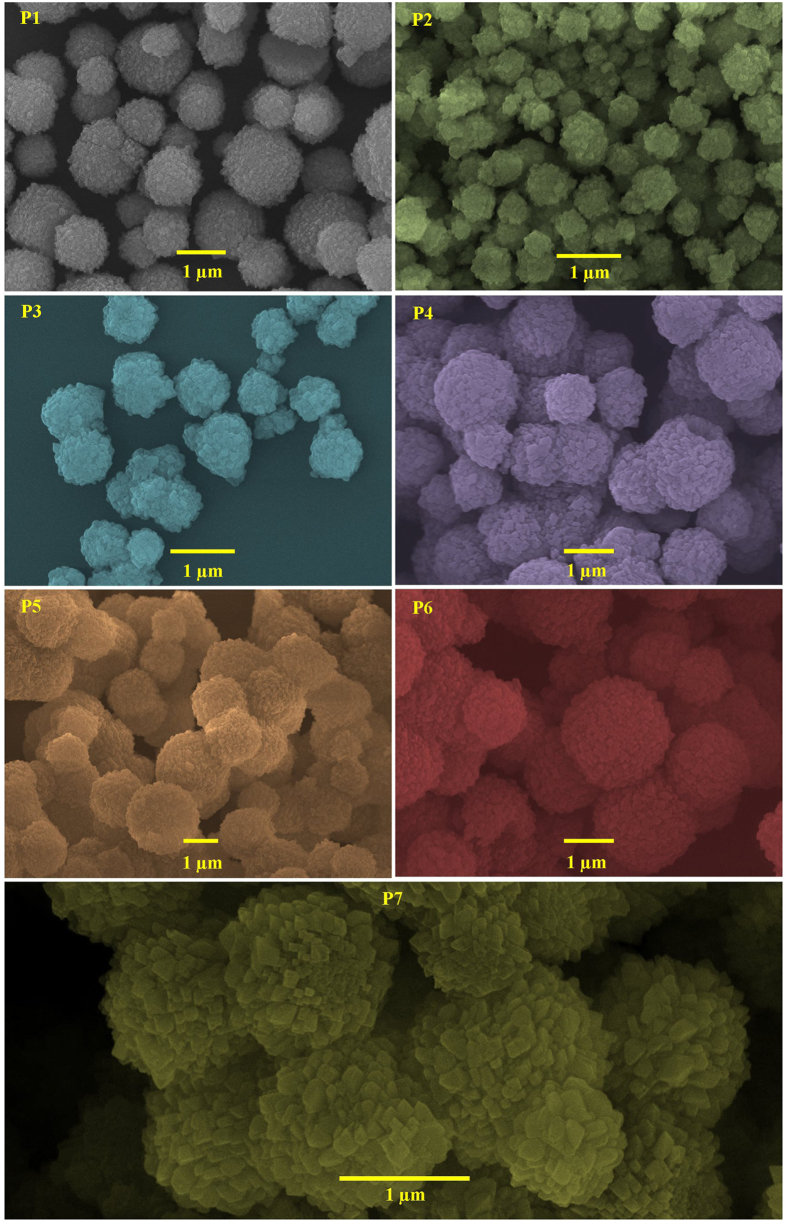
SEM images of knobby surfaced, microspheres of as-synthesized GIS-NaP1 zeolite microspheres with different Si/Al ratio.

Different magnification SEM images (Fig. 2 and supplementary information SI1) are used to identify and explain the effect of silicon content on the surface morphology, shape and size on GIS-NaP1 zeolite particles synthesis. SEM images of P1 sample reveal that GIS-NaP1 zeolite particles are well grown microspheres. These images also indicate that the microspheres have regular morphology and having size varying from 0.7–3.0 μm. The magnified images of P1 sample provided in supplementary information SI1 P1 indicate that GIS-NaP1 zeolite nano crystallites are well grown on microsphere surface and size of each crystallite is of few nano meters. In the second set of experiment when we increase the Si content during P2 synthesis surprisingly we obtained GIS-NaP1 particles of different shape, size and morphology in comparison to the earlier one. GIS-NaP1 zeolite sample P1 obtained from Si/Al ratio 5 shows well grown microspheres whereas sample P2 synthesized from Si/Al ratio 6 have crystals of irregular morphology. Beside, most of the P2 sample particles have size around 800 nm or less than 1 μm which is half of the size of P1 sample particles. The presence of nano crystallites in the vicinity of micron size GIS-NaP1 zeolite particles in the micrographs of P2 sample reported in supplementary information SI1P2 predicts that after certain point of crystallization nano crystallite grown enough and get detached from sphere’s surface. The distorted shape of GIS-NaP1 zeolite particles may be only because of fast growth of nano crystallites in P2 sample. We also noticed the broken sphere in the SEM images of P2 sample. Moreover, P2 sample does not look like spherical microspheres but instead of that these crystals appears to be micron size aggregates of nano crystallites. An increase in Si/Al molar ratio from 5 to 6 results in large morphological change of GIS-NaP1 zeolite microspheres, so it was expected that further increase in Si/Al ratio 6 to 7 may lead to complete destruction of GIS-NaP1 zeolite spheres and resultant product may have large volume of nano crystallites from which actual microsphere formed by oriented aggregation process, but in contrary, an improvement in the morphology, shape and size is observed in sample P3 with Si/Al ratio 7. The morphology of P3 sample particles is just similar to P2 sample particles except composed form of their nano crystallites (Fig. 2 and supplementary information SI1 P3). Afterward synthesized GIS-NaP1 zeolite samples having Si/Al ratio 8–11 have spherical morphology with well grown knobby surfaced microspheres. The SEM images of all these samples also predict some kind of aggregation in between the GIS-NaP1 microspheres. A thorough investigation of SEM images (Fig. 2 and supplementary information SI1) of sample P1 to P7 reveals that P5 sample’s particles have best morphology and least aggregation. The average particle size of GIS-NaP1 zeolite microspheres obtained from sample P5 is ~2 μm. The SEM results for the existence of nano-size particles were also found to be in good agreement with the crystallite size data and XRD diffraction pattern peak broadening obtained from the XRD study (Fig. 1).

**Figure 3:**
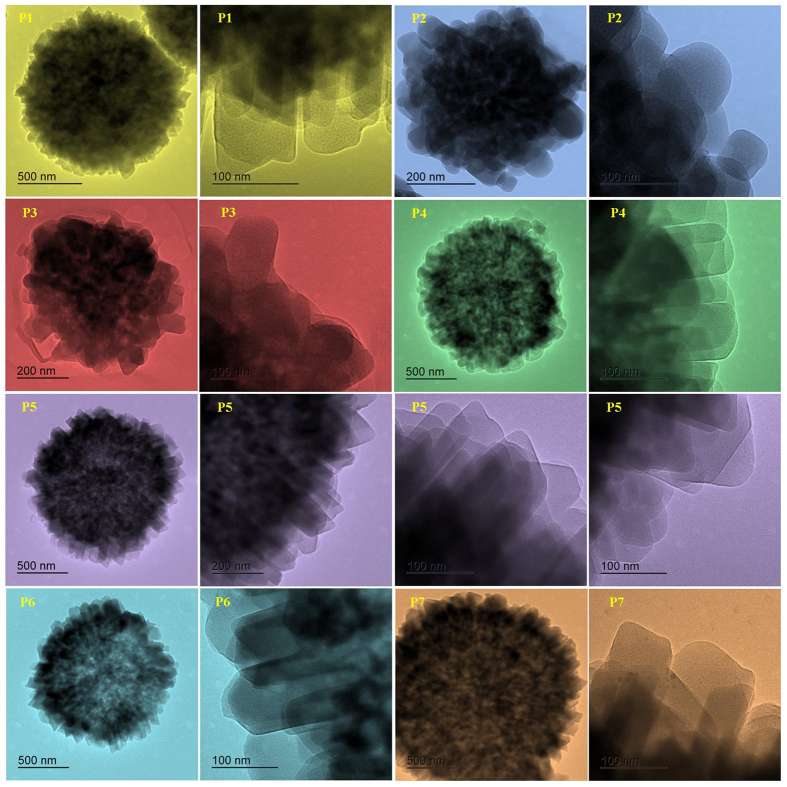
TEM images representing knobby surfaced microspheres and magnified view of nano crystallites of as-synthesized GIS-NaP1 zeolite crystals with different Si/Al ratio.

**Figure 4:**
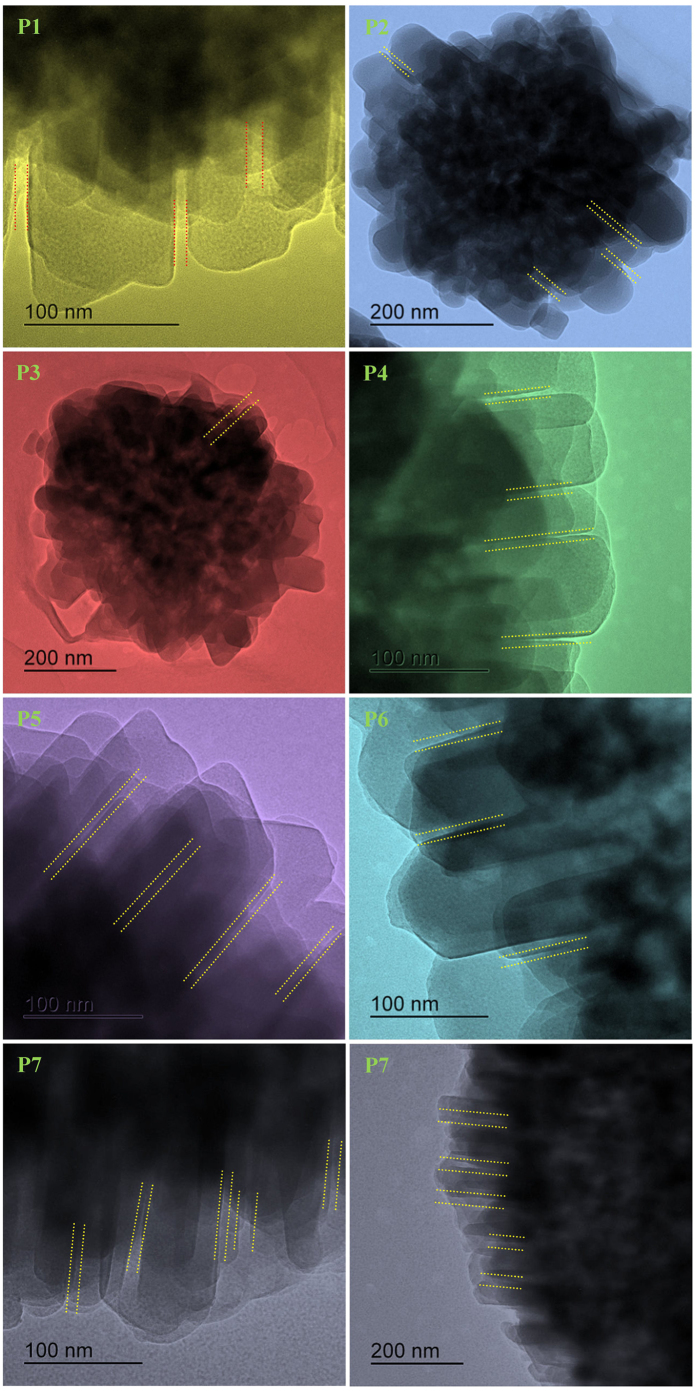
High magnification TEM images demonstrates that the Si/Al ratio affects the length and width of inter crystallite or intra microsphere void generated by the self-oriented arrangement of nano crystallite or mesoporosity of as-synthesized GIS-NaP1 zeolite crystals.

Further, to convince and show individual crystallite on GIS-NaP1 zeolite microsphere, TEM studies were performed. TEM inspection (Fig. 3) also confirms that silica content in reaction mixture plays a significant role in controlling the morphology of individual sphere as well as crystallites. On analysing the TEM images we find that except P2 and P3 samples, the growth of crystallites on GIS-NaP1 zeolite spheres is uniform in all other samples (P1, P4, P5, P6 and P7). Moreover, the magnified images of nano crystallites in sample P2 and P3 show that crystallites have round edges whereas all other samples have sharp edges, but sharpness of edges varies from sample to sample. As SEM analysis (Fig. 2) reveals that GIS-NaP1 zeolite sample P5 crystallites have highest uniformity in size and shape, similarly TEM results indicate the complete growth of crystallites with sharp edges. These TEM micrographs (Fig. 3) also demonstrate that the size of surface crystallites is comparatively bigger than the second and third layer crystallites and the shape of surface crystallites appear like rectangle/parallelogram with filleted edges. These TEM images (Fig. 3) also demonstrate that vertical arrangement of all these nano crystallite creates slit shape micropores. TEM images reported in Fig. 4 reveal Si/Al ratio have paramount effect on the morphology, size and shape of nano crystallites which ultimately affects the microporosity of GIS-NaP1 zeolite samples.

As Si/Al ratio is the key for maximizing adsorption performance, therefore, selected area electron diffraction studies were performed on each GIS-NaP1 zeolite samples obtained from different Si/Al ratio reaction mixture. The chemical composition in tabular form of the GIS-NaP1 zeolite samples are presented in Table 1. The main objective of the present study is to investigate the effect of Si content on the purity, performance and morphology of GIS-NaP1 zeolite, therefore, detail elemental analysis was made. Since it is clear from XRD (Fig. 1) and SEM (Fig. 2) analysis that in the specimens, no crystalline impurities are present, therefore reported composition will be of phase pure GIS-NaP1 zeolite microspheres. Table 1 represents the chemical composition of knobby surface GIS-NaP1 zeolite microspheres. The summarized elemental analysis data in terms of weight and atomic percentage of all the selected area reveals that the framework Si/Al ratio of final product increases with increase in the initial silica content till Si/Al ratio reaches 10. But after that further increase in Si content does not results in the increase of Si/Al framework composition. On comparing Si/Al ratio of each of the selected area sample we found that in P6 specimen the knobby surface sphere have Si/Al ratio 2.34 (Table 1) whereas P1 and P7 have 1.65 and 2.11, respectively. The EDS analysis results of GIS-NaP1 zeolite samples reveal a noticeable variation in Si/Al ratios for some of the samples. Furthermore, EDS results of P6 sample indicate that the Si/Al ratio is quite high in comparison to P1 sample.

**Figure 5:**
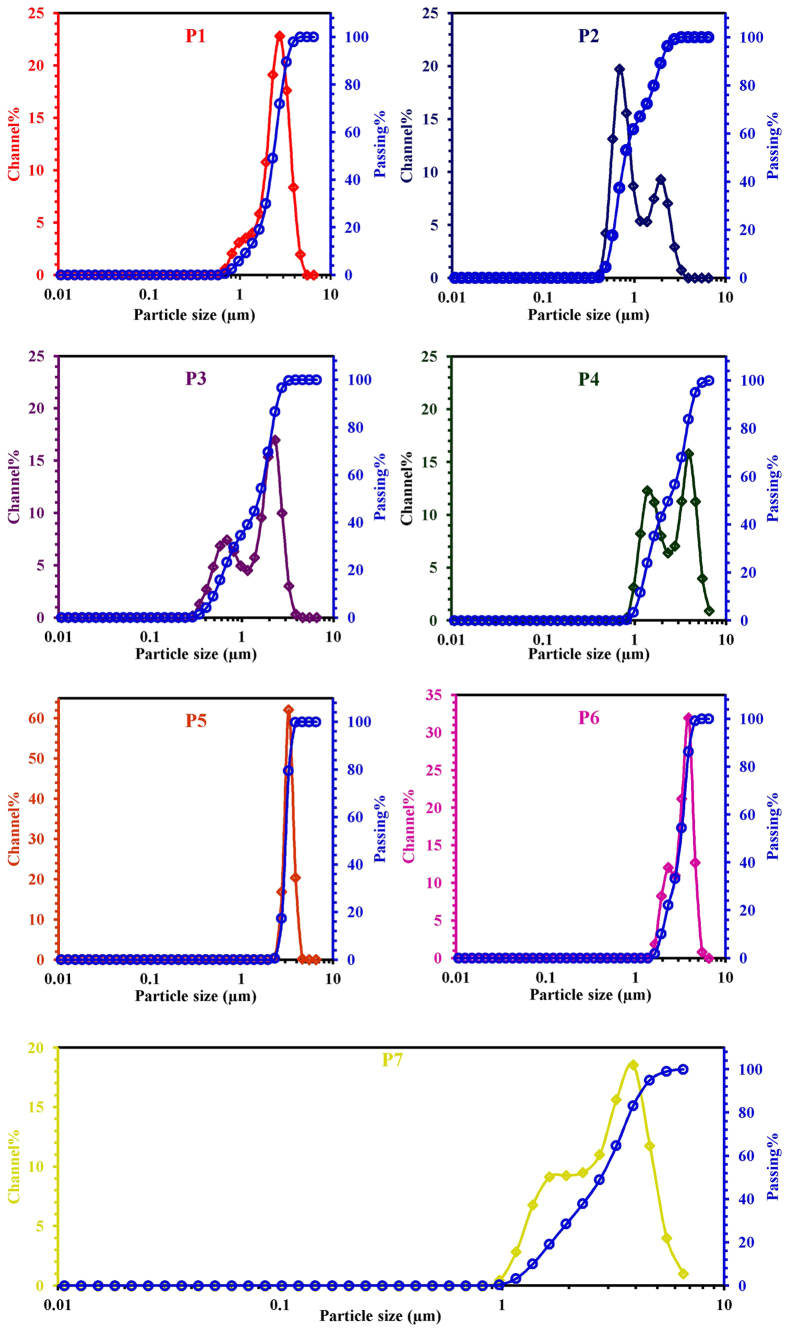
Curves representing the particle size distribution of as-synthesized GIS-NaP1 zeolite microspheres having different Si/Al ratio.

From the compiled LSA data an attempt is made to clearly understand the existence and formation of particles having different size, shape and morphology caused by different phenomenon such as agglomeration, crystallization, phase changes, crystal packaging etc., occurring during crystal growth process. The PSD curve of P1 sample shows well distinguished population of GIS-NaP1 zeolite particles with a mean size of ~2.75 μm and a very little fraction of nano crystals (Fig. 5). The presence of nano size particles in large volume is observed in P2 zeolite sample which confirms that particle size of GIS-NaP1 zeolite sample obtained from Si/Al ratio 6 is quite low than that of P1 sample. The PSD curve of P3 sample is just reverse of P2 sample i.e., volume of micron size particle is more that nano size (Fig. 5). The light scattering analysis of the P4 sample shows the presence of microspheres with wide size distribution. Surprisingly, for sample P5 having Si/Al ratio 9 in the initial reaction mixture, we do not find any sign of aggregation and existence of nano size particles (Fig. 5). The PSD curve of P5 zeolite sample indicates the formation of well distributed, similar sizes GIS-NaP1 zeolite knobby spheres of size around 3 μm. Whereas the PSD data of samples P6 and P7 exhibits increase in size and particle size distribution with increase in Si content in the reaction mixture. Although LSA studies provide just primary information about the nature and size of particles but the PSD results are in good agreement with SEM observations. The electron micrographs (Fig. 2) and light scattering results convincingly show that the size, shape and morphology of GIS-NaP1 zeolite crystals are highly dependent on Si/Al molar ratio used for hydrothermal reaction mixture. The results summarized in Figs 2 and 5 demonstrate that with the increase in Si/Al ratio (>6) particle size keep on increasing from nano size to micron size. LSA data also concludes the existence of some aggregation which has also been earlier interpreted by SEM micrographs (Fig. 2).

**Figure 6:**
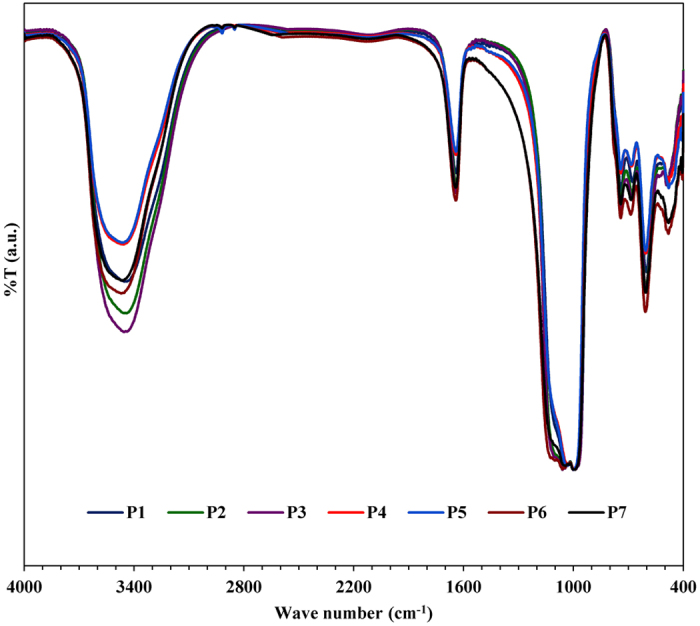
FTIR spectra of as-synthesized GIS-NaP1 zeolite microspheres having different Si/Al ratio.

GIS-NaP1 zeolite samples obtained from reaction mixtures having different Si/Al molar ratio were examined by FT-IR to complete long-range order XRD studies with short-range FT-IR information. Figure 6 collectively presents the FT-IR spectra of all GIS-NaP1 zeolite samples which match with each other and also to the reported infrared spectral data for zeolite P^27^,^28^. Furthermore, no impurity peak was observed in any of the infrared spectrum of GIS-NaP1 zeolite samples. In the collective FT-IR spectrum, the band around 605 cm^−1^ corresponds to the presence of double ring in the GIS-NaP1 zeolite framework and an intense band around 990 cm^−1^ along with week shoulder ~1100 cm^−1^ corresponds to T-O-T asymmetric stretching vibration. Intense and sharp bands around 490 cm^−1^ may be related to T-O bending mode. In addition, band appearing at around 745 cm^−1^, most probably, could be attributed to symmetrical stretching vibrations related to external linkages of TO_4_ units in zeolite structure. The bands around 3450 and 1640 cm^−1^ are the characteristic peaks of hydrate water in solid phase. Therefore, FT-IR analysis assures that the final product have only GIS-NaP1 phase and complement the results obtained from XRD, SEM and EDS studies.

### Water vapour adsorption

**Figure 7:**
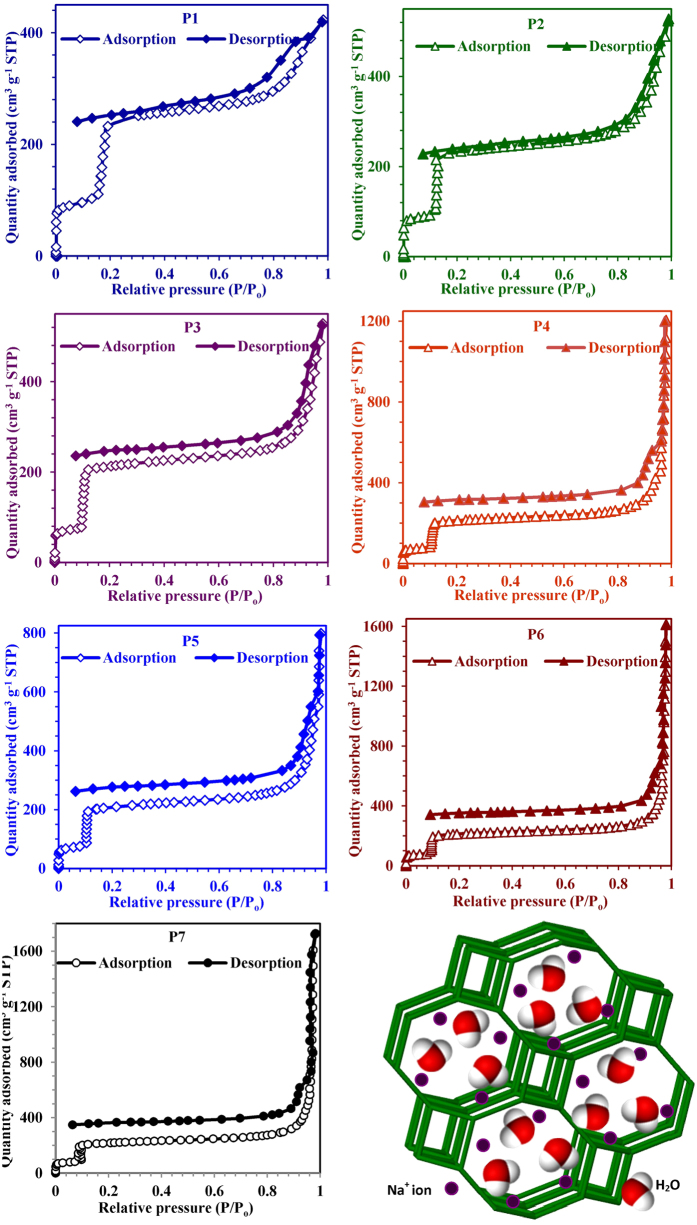
Water vapour adsorption-desorption isotherms of GIS-NaP1 zeolite microspheres obtained from different Si/Al ratio precursors reaction mixture at 298 K, and GIS-NaP1 zeolite framework.

Water vapour adsorption isotherms of phase pure GIS-NaP1 zeolite are presented in Fig. 7. At 298 K, the water vapour adsorption on GIS-NaP1 zeolite displays a characteristic S-shaped isotherm which also referred to as type VI (instead of type IV)^29^. The characteristic S-shaped isotherm is mainly caused by the presence of different potential adsorption sites (i.e. channel interactions and interiors) and by an intricate panel of adsorbate-adsorbate interactions^30^. According to Sastre and Corma, the adsorption potential of zeolites is strongly influenced by the confinement level of the sorbate molecules in the zeolite matrix^31^. The “confinement effect” is mainly caused by the internal surface curvature of zeolite pores, promoting nonspecific interactions between the oxygen atoms in the zeolite framework and the molecules located in their intra-crystalline free space. The confinement effect also tends to optimize van der Waals interactions in zeolite cavities involving a perturbation of the shape and electronic structure of the adsorbate. This effect is magnified for tight-fitting adsorbate molecules in zeolitic pores. The confinement effect of zeolites is at the origin of adsorbate induced framework structural changes observed for some zeolite/adsorbate systems^32^,^33^,^34^. Furthermore, these isotherms reveal the high water vapour adsorption capacity of these GIS-NaP1 zeolite samples. The quantity of water vapour adsorbed on high silica GIS-NaP1 zeolites (P7 = 1728 cm^3^ g^−1^ and P6 = 1616 cm^3^ g^−1^) is much higher than the low silica samples (P1 = 423 cm^3^ g^−1^ and P2 = 528 cm^3^ g^−1^). As Gatta *et al.*^35^ reported that in the channels of GIS zeolite, each Na^+^ ion is coordinated with four oxygen atoms and two water molecules (Schematically presented in Fig. 7), the water content in the GIS zeolite samples was approximately 17 weight percent which corresponds to 12 water molecules per unit cell. So, the high water vapour adsorption on the as-synthesized GIS-NaP1 zeolite samples is justifiable and there is also a possibility of water condensation on zeolite surface during adsorption process. Sometime, the existence of different types pores in the adsorbent materials cause Type VI adsorption isotherms. As we have seen in the SEM and TEM micrographs of GIS-NaP1 zeolite samples (Figs 2, 3, 4) that each microsphere is composed of large number of nano crystallites, therefore there is also possibility of inter-crystallite void formation and these inter-crystallites voids can also play a vital role in water vapour adsorption. Apart from S shape adsorption isotherm and high water vapour adsorption tendency, the other important point unveiled by this work is that even at relative pressure of 1, there is no sign of saturation instead a sharp water vapour adsorption was observed. As these GIS-NaP1 zeolite samples show high water vapour adsorption capacity, therefore, we decided to compare their water uptake tendency with some potential water vapour adsorbents (LTA and FAU type zeolites)^17^,^18^,^19^,^20^,^21^,^22^,^23^.

**Figure 8:**
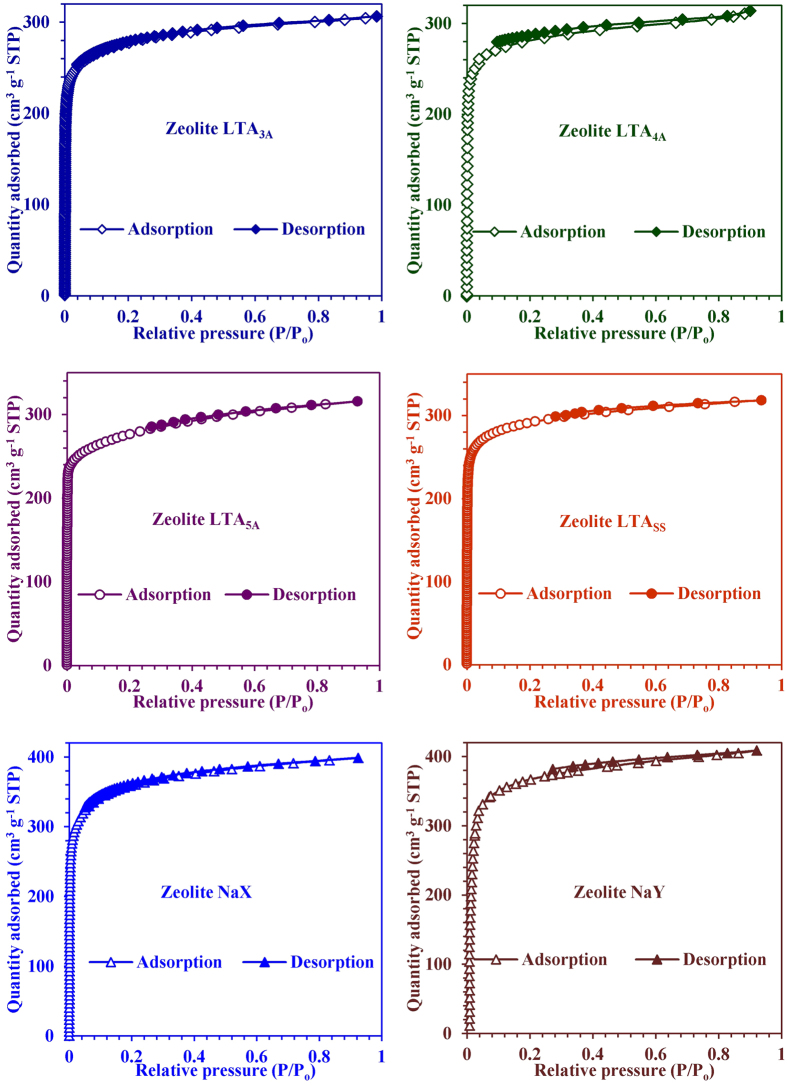
Graphs represent the water vapour adsorption-desorption isotherms of commercial LTA (3A, 4A and 5A), NaX, NaY, and self-synthesized (SS) LTA zeolite samples.

**Figure 9:**
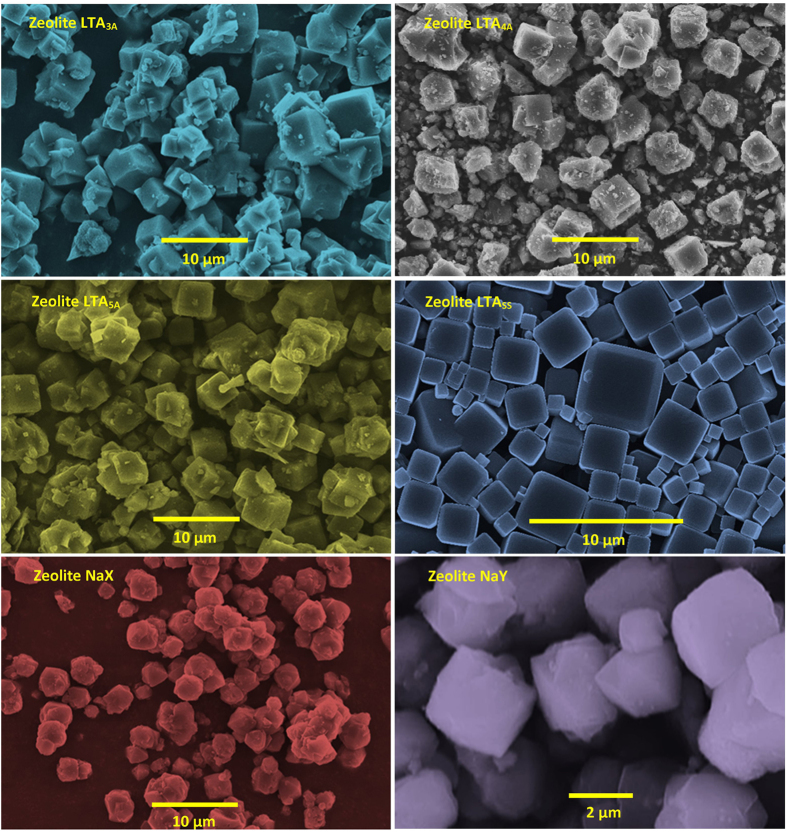
SEM images of commercial LTA (3A, 4A and 5A), NaX, NaY, and self-synthesized LTA zeolite crystals those used for comparative water vapour adsorption studies.

To make a healthy comparison, we first studied the water uptake properties of commercially available molecular sieves 3A, 4A, 5A (LTA zeolite), NaX, NaY and self-synthesized LTA zeolite on the same water vapour adsorption instrument (BELSORP-max, BEL Japan, Inc.) that was used for GIS-NaP1 zeolite samples. The obtained water vapour adsorption data for all these commercial adsorbents and LTA_SS_ zeolite have been plotted in Fig. 8 whereas SEM images are reported in Fig. 9. All these adsorption isotherms (Fig. 8) exhibit a Type I shape in the IUPAC classification, characteristic of the adsorption on microporous solid. The isotherms reveal that these commercial microporous zeolites captures water vapours at very low P/P_o_ value with steep uptake behaviour because of their greater affinity for water. The nature of these isotherms and water uptake values are also comparable with the data reported by other researcher^3^,^18^,^19^,^20^,^21^,^22^,^23^. But on comparing these isotherms with GIS-NaP1 zeolite samples we found that both are totally different from each other. The earlier isotherms (Fig. 7 GIS-NaP1 zeolite samples) are of S shape (or Type VI) whereas later of Type I. However, GIS-NaP1 zeolite samples show limited water uptake (~115 cm^3^ g^−1^) in comparison to LTA (~280 cm^3^ g^−1^) and FAU (~350 cm^3^ g^−1^) type zeolite at lower pressure (P/P_o_ < 0.1) which indicates relatively low affinity of GIS-NaP1 zeolite surface for water molecules. This is related to low hydrophilicity of GIS type zeolite in comparison to LTA and FAU type zeolites. Moreover, the pore diameter of GIS type zeolites is relatively small so it can also affect the easy occupancy of zeolitic micropores. S shape isotherms and relatively high water uptake at P/P_o_ = 0.3 indicate the presence of mesoporous (non-zeolitic pores) as well as microporous or the presence of different potential adsorption sites. The high water adsorption capacity (423.4–1727.5 cm^3^ g^−1^) and steep water uptake tendency of GIS-NaP1 zeolite sample at high relative pressure indicates the meso-macropore filling and condensation of water vapours into the pores as well as on nano crystallites surface. The rare Type VI isotherm is also a characteristic of hydrophilic adsorbent, and indicative of multilayer adsorption. The water adsorption-desorption isotherms for GIS-NaP1 zeolite samples (Fig. 7) display significant steps with hysteresis loops. Limited water uptake of as synthesized GIS-NaP1 zeolite samples at low pressure in comparison to highly hydrophilic LTA and FAU type zeolite samples indicates their low hydrophilicity. Therefore, their higher water uptake is closely related to their meso as well as macroporous character (inter microsphere voids). Also it has reported that even mesoporous hydrophobic materials (SBA-15, MCM-41, MOFs, carbon based materials etc.) show even higher water adsorption capacity than that of hydrophilic zeolites and silica gels (SGs)^36^. Therefore, a hydrophilic material is not necessary to have a high water uptake capacity, whereby the water uptake capacity is mainly determined from the pore volume, pore size and pore structure of the materials. In fact, the hydrophilicity of an adsorbent is defined by its selectivity to water compare to other adsorbates at particular pressure.

**Figure 10:**
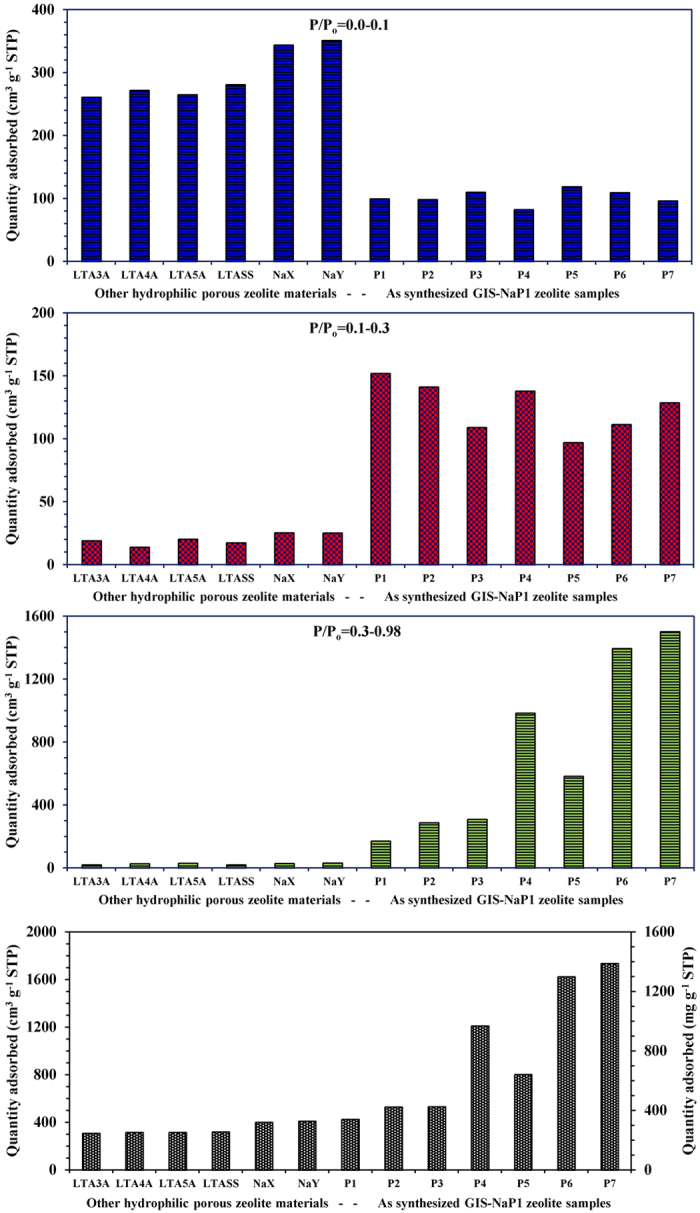
Comparison of water vapour uptake capacity of other hydrophilic porous zeolite materials and as-synthesized GIS-NaP1 zeolite samples in different pressure ranges (P/P_o_), and total water vapour adsorption capacities.

As different applications require different operational or optimum pressure such as water adsorption chiller/water capture (P/P_o_ ≤ 0.1), temperature triggered water capture-release system (P/P_o_~0.3) etc.^18^. Therefore, the water vapour adsorption capacity of all studied adsorbents at various ranges of relative pressures P/P_o_ has reported in Fig. 10 as bar graphs. The commercial NaX and NaY show maximum water uptake around 28 wt.% at P/P_o_ = 0.1 which is higher than any other zeolite material. On the other hand, GIS-NaP1 zeolite sample show water uptake of around 10 wt.% in the relative pressure range P/P_o_ = 0.1–0.3 whereas all other zeolite materials (LTA and FAU) report negligible adsorption in this pressure range. The isotherm profile as well as bar graph (Fig. 10) of GIS-NaP1 zeolite materials demonstrate significant amount of water vapours uptake (P1–P7 = 14–120 wt.%) in the pressure range P/P_o_ = 0.3–0.98. The highest water vapour adsorption capacity of 1727.5 cm^3^ g^−1^ is observed for P7 zeolite sample. To the best of our knowledge, this is the highest value of water uptake at relative pressure of 0.98.

**Figure 11:**
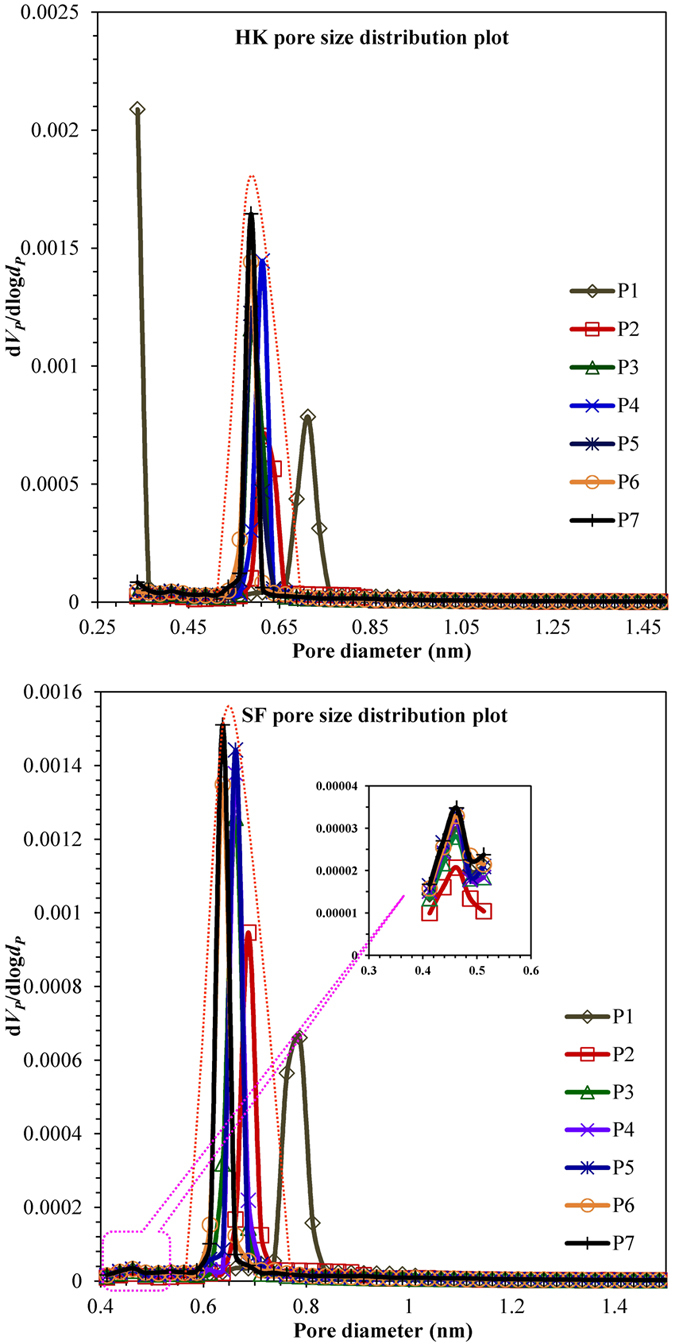
Water vapour adsorption HK and SF pore size distribution curves for GIS-NaP1 zeolite samples synthesized from different Si/Al ratio precursors reaction mixture. Inset image indicates the existence of some smaller pores.

Table 2 lists the BET surface area, structural parameters and water vapour adsorption capacities of different zeolite materials. The monolayer Langmuir surface area of GIS-NaP1 zeolite samples is found higher in comparison to other zeolite types materials reported in the present work. The pore size distribution curves obtained from HK and SF analysis methods (Fig. 11) indicate the microporous character with narrow pore size distribution. All the material except sample P1 show uniformity in the pores as per HK as well as SF plot. These graphs indicate that although there is significant variation in the Si/Al ratio of as-synthesized GIS-NaP1 zeolite samples but difference in the mean diameter of the micropores is relatively small. With this water vapour adsorption study we cannot confirm that the reported values correspond to GIS-NaP1 zeolite pores or secondary non-zeolitic pores formed during the oriented aggregation process of primary GIS-NaP1 zeolite nano crystallites. In general, it is always difficult to find the exact pore size of materials having pore diameter <0.3 nm and GIS framework type zeolite have a narrower pore entrance compared to other zeolite types. So, the reported micropore sizes cannot be considered as true value as in literature reported size of GIS-NaP1 zeolite pores is around 0.29 nm^1^,^28^. Apart from the water vapour adsorption isotherms (Fig. 7), these adsorption data (HK and SF method i.e., mathematical relationship between relative pressure and pore size) predicts the microporous as well mesoporous character of the reported materials. Furthermore, on comparison the HK and SF pore distribution plots of GIS-NaP1 zeolites samples (Fig. 11) (having theoretic pore diameter 0.29 nm) with the LTA (~0.40 nm) and FAU (~0.74–1.10 nm) zeolites samples (supplementary information SI3), we reconnoitred that earlier samples have complete graphical curves (Fig. 11, highlighted by dotted lines) whereas later show half curves (supplementary information SI3). A close look of these graphical curves reveals that the deviation in pore volume with the change of pore diameter (dV_P_/dlogd_P_) is much higher for GIS-NaP1 zeolite samples, which also indicates the existence of some non-zeolitic micropores along with zeolitic structural pores. Therefore, considering an importance of pore structure/porous character of an adsorbent for water adsorption capacity, N_2_ gas adsorption studies were made on the as-synthesized GIS-NaP1 zeolite samples.

### Physical adsorption characteristics of miro-meso-macroporous GIS-NaP1 zeolite microspheres

**Figure 12:**
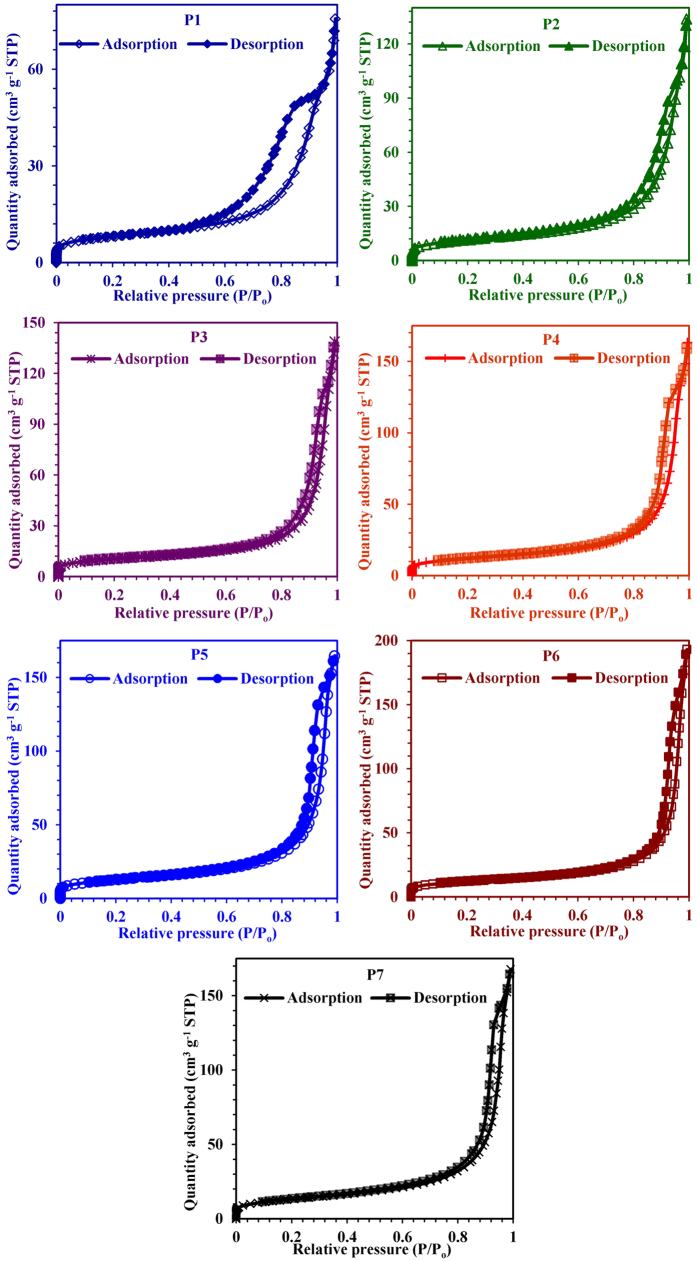
N_2_ gas adsorption-desorption isotherms of GIS-NaP1 zeolite microspheres obtained from different Si/Al ratio precursors reaction mixture at 77 K.

**Figure 13:**
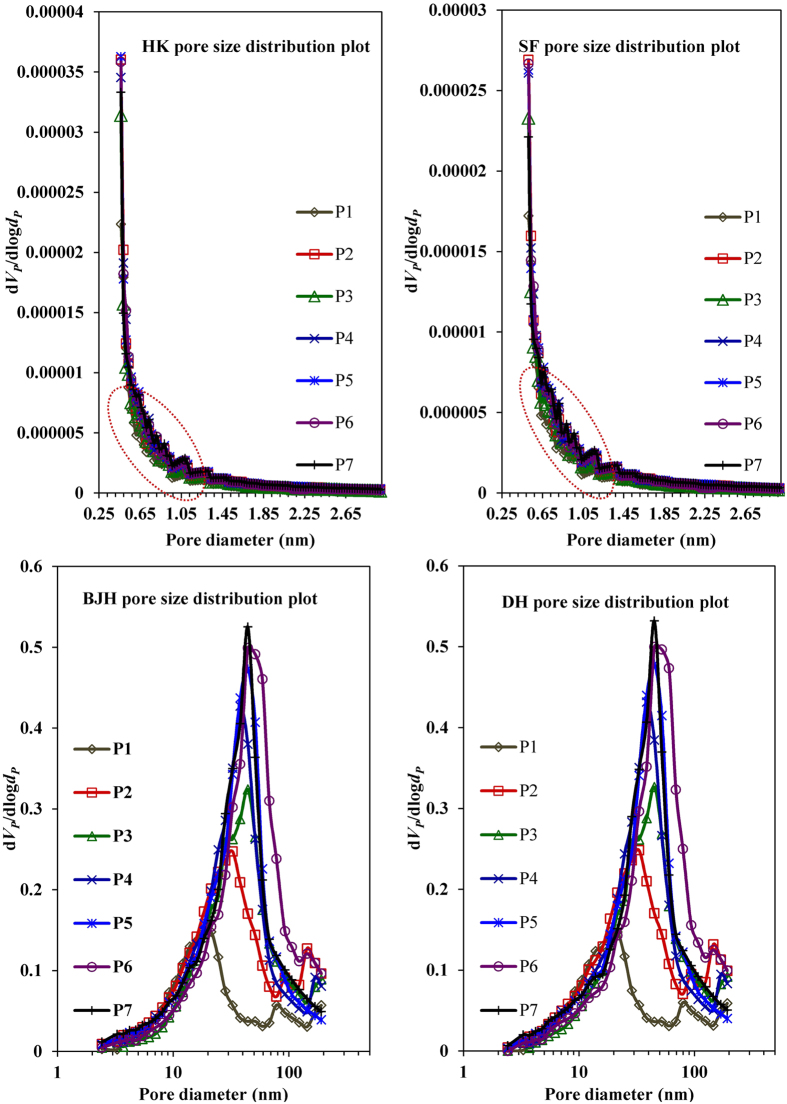
HK, SF, BJH, and DH pore size distribution curves obtained from N_2_ gas adsorption for GIS-NaP1 zeolite samples synthesized from different Si/Al ratio precursor’s reaction mixture.

As in the previous section we explored wondrously high water uptake by GIS-NaP1 zeolite microspheres which obligate us to further explain these findings by discussing the various porous features (micro-meso-macroporous pore distributions) by comparing different pore characterisation methods based on the analysis of N_2_ adsorption isotherms at its boiling point. The effect of Si/Al ratio on the porous or textural properties will be the focus of this discussion. Figure 12 shows N_2_ adsorption-desorption isotherms at 77 K of GIS-NaP1 zeolite samples prepared from different Si/Al ratio precursors’ solutions while the main textural parameters obtained by applying different methods are compiled in Table 3. The porosity of the GIS- NaP1 zeolite microspheres displays a Type IV isotherm with prominent hysteresis loop in the desorption branch at high relative pressure which further indicates the characteristic of a meso-macroporous material and larger inter-particle porosity in the products. These hysteresis loops which look somewhat like inverse type H2 or H3 hysteresis have been associated with the occurrence of pore blocking, wide distribution of independent pores etc. Inverse type H2 hysteresis has been observed for instance in meso-macroporous materials, cavities/channels with several characteristic sizes, or in materials where the entrances to the spherical pores had been widened by hydrothermal treatment. This behaviour can, for instance, be caused assemblages of slit-shaped pores or by the existence of non-rigid aggregates of plate-like particles^37^. In these case, the distribution of necks/constrictions is much wider than the distribution of main pore cavities, therefore, the adsorption/condensation branch is steeper than the desorption branch. Hence, the distribution of neck sizes can be obtained from the analysis of desorption branch whereas the pore/cavity size distribution is only available from an analysis of the adsorption branch (e.g., by applying a method for pore size analysis which correctly takes into account the delay in condensation such as the NLDFT). Low BET specific surface area values for all these GIS-NaP1 zeolite samples reported in Table 3 also demonstrate the existence of macropores character of the same. Furthermore, low slope region in middle of isotherm indicates first few multilayers on external surface including meso and macropores before the onset of capillary condensation. Although N_2_ adsorption is considered to be a standard adsorptive for surface area and pore analysis but it is generally accepted that nitrogen adsorption is not satisfactory with regard to the assessment for relatively small micropores such as 8 membered rings of size ~0.29 nm in case of GIS-NaP1 zeolite. Therefore, the average micropore size values quoted in Table 3, and HK and SF-micropore size distribution curves (Fig. 13) could not be considered as actual or accurate values. However, the starting point of HK and SF-micropore size distribution curves (Fig. 13) reveal the existence of microporosity of the studied materials. A close watch of BJH and DH mesopore size distribution curves (Fig. 13) indicates the existence of large volume of meso (2–50 nm) as well as macropores (>50 nm). These pore distribution curves reveal that with the increase in Si/Al ratio, shifts in the peak positions toward high pore value have been observed. Further, a surprising assessment is made that the peak intensities of sample P1, P2 and P3 are quite low in comparison to other samples, and have bimodal curves, which indicates that these materials have low mesoporous character and non-uniform aggregation of secondary particles (microspheres). These meso- and macropores were definitely generated due the oriented aggregation phenomenon of primary nano-crystallites and secondary microspheres. The aggregation behaviour has been well established by the SEM (Fig. 2), TEM (Figs 3 and 4) and LSA (Fig. 5) studies, and these are in good agreement with pore size distribution results. Mesopore values reported in Table 3 are just an average value of pores distribution (meso as well macro) obtained from BJH plot (Fig. 13) for each sample.

**Figure 14:**
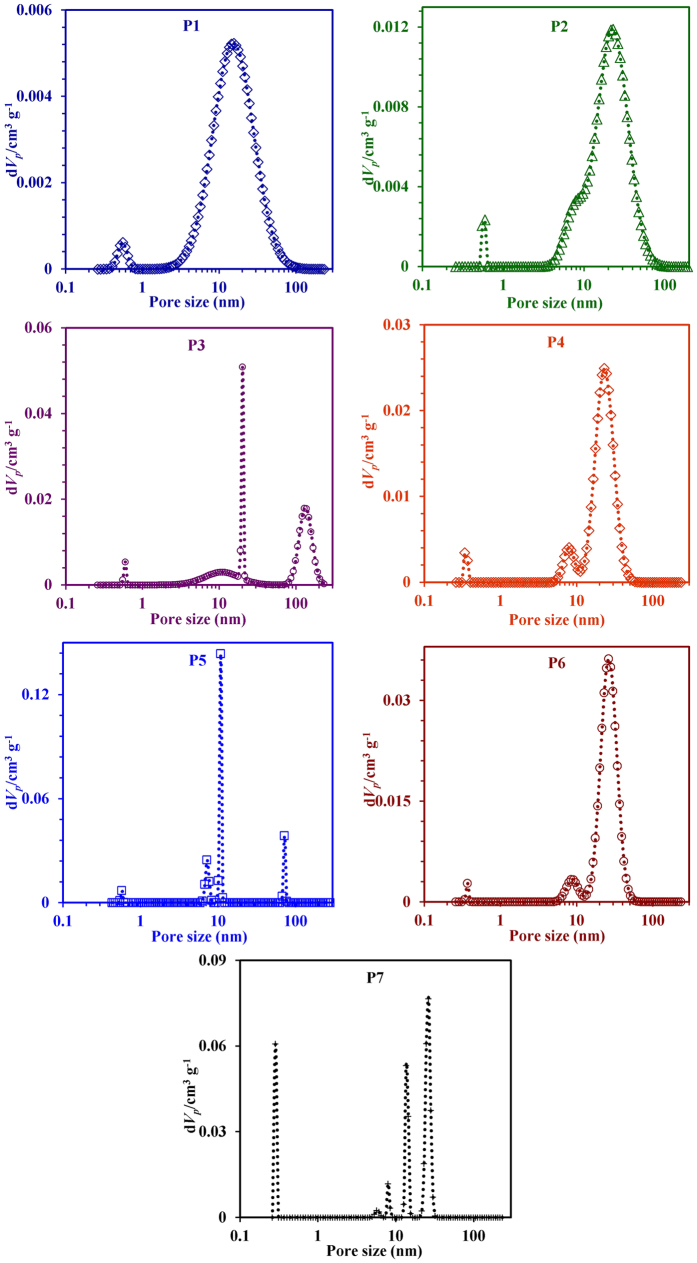
NLDFT pore size distributions of the GIS-NaP1 zeolite samples derived from N_2_ adsorption isotherms.

NLDFT methods which allow one to obtain a reliable pore size distribution from the adsorption branch are crucial for the pore size analysis of materials in complex pore networks. The pore size analysis results obtained from NLDFT method and drawn in Fig. 14 agree very well with other independent electron microscopic and scattering analysis techniques. These pore size distribution curves confirm the multistep adsorption behaviour of water vapours on micro-meso-macroporous GIS-NaP1 zeolite microspheres, and detailed description has been reported in final section of manuscript. The pore size distribution curves corresponding to low Si/Al ratios samples P1 and P2 (Fig. 14) are bimodal and second distribution curve predicts the wide distribution of mesopores whereas the volume of micropores is quite low. On the other hand, P3, P4, P5 and P6, samples have trimodal, and P7 tetramodal behaviour. NLDFT curve for high silica sample P7 reveals that this GIS-NaP1 zeolite sample has similar and narrow distribution of micro-, meso- and macropores. On comparing NLDFT (Fig. 14) data with LSA data (Fig. 5) and SEM micrographs (Fig. 2 and supplementary information SI1) we found that the increase in aggregation of secondary microsphere meso- and macroporosity was also increased whereas the crystallinity of the materials effects their pore distribution. NLDFT curve of sample P5 (Fig. 14) shows maximum mesopores having pore diameters <10 nm which may just corresponds to the inter-crystallite pore or voids generated by the nano-crystallites those were accumulated by GIS-NaP1 zeolite microspheres, because particle size distribution graph for same sample reported in Fig. 5 show narrow distribution or no aggregation. Therefore, change in the porosity of material is entirely dependent on the shape, size, crystallinity and aggregation behaviour of nano crystallite as well as the microspheres i.e., inter-particle spacing.

### Measurement of water uptake, hydrophobicity and thermal stability by thermogravimetry method

**Figure 15:**
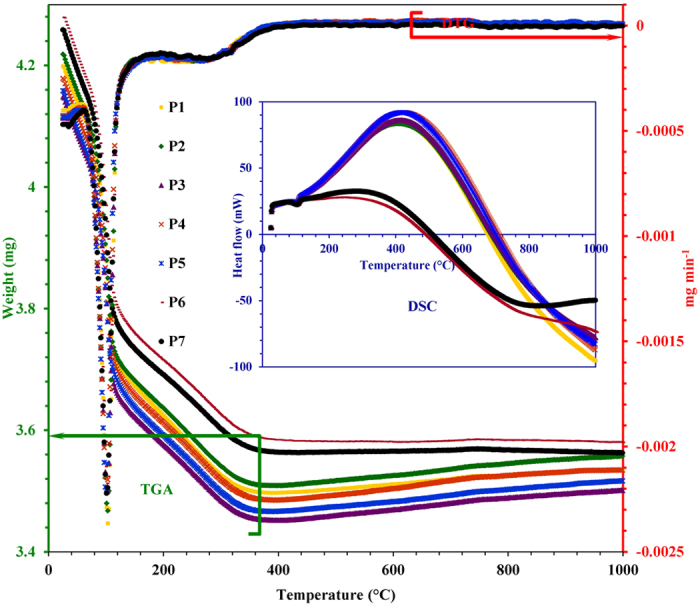
Thermo-gravimetric analysis (TGA), derivative thermogravimetry (DTG) and differential scanning calorimetry (DSC) curves of the GIS-NaP1 zeolite samples using a temperature ramp of 10 °C min^−1^.

TGA, DTG and DSC curves for GIS-NaP1 zeolite samples are collectively presented in Fig. 15 whereas the various thermograms for commercial samples (LTA and FAU type zeolites) are presented as supplementary information SI4. TGA and DTG curves represent the two step dehydration i.e., fast desorption of surface water or weakly attached water molecules in the temperature range 25–100 °C, and further weight loss in the temperature range 100–400 °C attributed to desorption of the remaining water enclosed/trapped in the material matrix, voids, channels etc., and progressive dehydration of material (physically/chemically adsorbed water). As per thermal stability is concerned P6 and P7 show highest thermal stability, even more than that of commercial molecular sieves reported in this paper (supplementary information SI4).

As nowadays several quantitative as well as qualitative techniques has been employed to study the water adsorption potential of the materials, therefore, along with BET analysis TG method has also been used to calculate HI and water adsorption capacity by dividing the weight loss due to water desorption to the weight of dried adsorbent. The calculated HI and water adsorption capacity values for as-synthesized as well as commercial zeolite samples are tabulated in Table 4. The obtained HI values show that GIS-NaP1 zeolite samples have moderate hydrophilicity as their HI values ~0.75 are comparable to LTA zeolite samples (HI = 0.60–0.71) which is considered to be highly hydrophilic zeolitic material based on high Al concentration. On comparing the water uptake capacities, we found FAU type zeolite material shows better adsorption capacities even more than that of LTA type zeolite. This is only because of the larger zeolitic pore diameters of FAU type zeolite (0.71–1.10 nm) than that of LTA (~0.40 nm) and GIS-NaP1 (~0.29 nm). This indicates that hydrophilic material is not necessary to have a high water adsorption capacity. Instead the water adsorption capacity is mainly determined from the pore structure, pore size, pore volume etc., of the materials. Water uptake capacity values reported at relative high pressure (Table 4) indicate that GIS-NaP1 favour multilayer adsorption and cavity filling by capillary condensation process whereas other reported commercial zeolite show (LTA and FAU) micropore filling at relative low pressure.

### Multistep water adsorption on micro-meso-macroporous GIS-NaP1 zeolite microsphere

**Figure 16:**
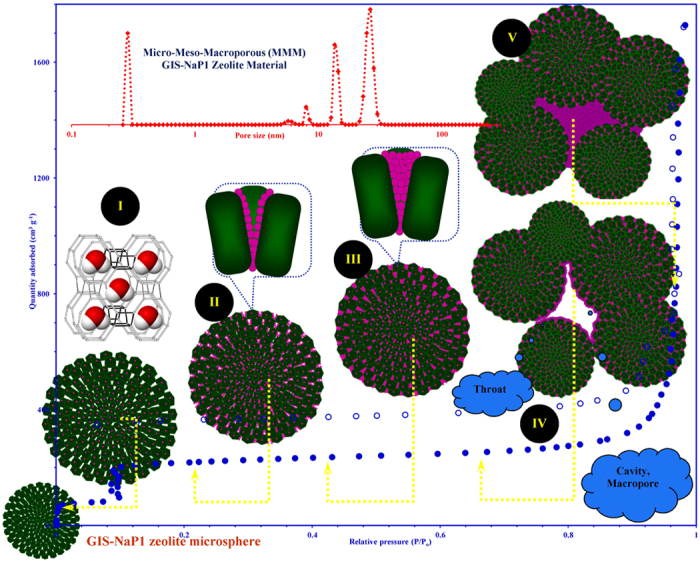
Graphical representation of multistep water vapour adsorption on micro-meso-macroporous GIS-NaP1 zeolite microsphere sample.

Based on all the observations an attempt is made through a graphical representation (Fig. 16) to explain the different adsorption steps those were took place on micro-meso-macroporous GIS-NaP1 zeolite microsphere sample during water vapour adsorption at various relative pressures. *Step I* indicates the accumulation of water vapours by extremely small zeolitic micropores (0.29 nm) and micropore filling ~11 wt.% water uptake. This indicates the relatively low affinity of zeolitic surface for water molecule and very narrow pore openings. To some extent it also relates moderate hydrophilicity of GIS-NaP1 zeolite. *Step II* corresponds to monolayer formation and mesopore filling. The high water uptake (~18 wt.%) in the relative pressure range P/P_o_ = 0.1–0.3 makes these material suitable for many thermal driven adsorption-desorption applications. This water uptake generally corresponds to the monolayer water vapour adsorption on the walls of slit/cylindrical shape voids generated by the regular arrangement of nano crystallites. Water uptake in this region also corresponds to the filling of inter-crystallite or intra-microsphere non-zeolitic pores. Even at low pressure water vapour adsorption on as-synthesized GIS-NaP1 zeolite has composed of multiple processes and adsorption can also be correlated to the filling of wide range of meso sized cavities/voids. The steep adsorption step at P/P_o_ = 0.1 confirms the start of mesopore filling. *Step III (multilayer development)*, once the water molecule adsorbed on the pore or cavity wall, water-water interaction take place via hydrogen bonding and water cluster as a single layer (*Step II*). These water clusters grow until a certain pressure and then capillary condensation occurs followed by filling of mesopores with water^5^. *Step IV* and V, nearly eight fold increase in the water uptake capacity at high relative vapour pressure (P/P_o_ ∼ 0.9) clearly illustrates that higher vapour pressure is required to induce the macropore filling. As shown in Fig. 16, *Step IV* and *V* might be attributed to monolayer followed by multi-hydrated layer growth into to an empty space after the mesopores were completely filled (P/P_o_ > 0.6), i.e., cavities and throats generated by the aggregates of GIS-NaP1 zeolite microspheres. This expeditious change in the water uptake by GIS-NaP1 microsphere involving the formation of multi-layers of adsorbed water molecules makes the data interpretation much more complex. So, apart from these water clusters formation, and capillary condensation based macropore filling, the other possible phenomena are: physisorption on GIS-NaP1 microspheres, hydration of surface cations, rehydration of surface, and rehydroxylation^5^. Moreover, solvation properties of exchangeable cations and possible change in the surface areas of aggregated zeolite materials during an increase in the relative pressure make the adsorption system more complex which eventually leads to multi-step adsorption^38^.

**Figure 17:**
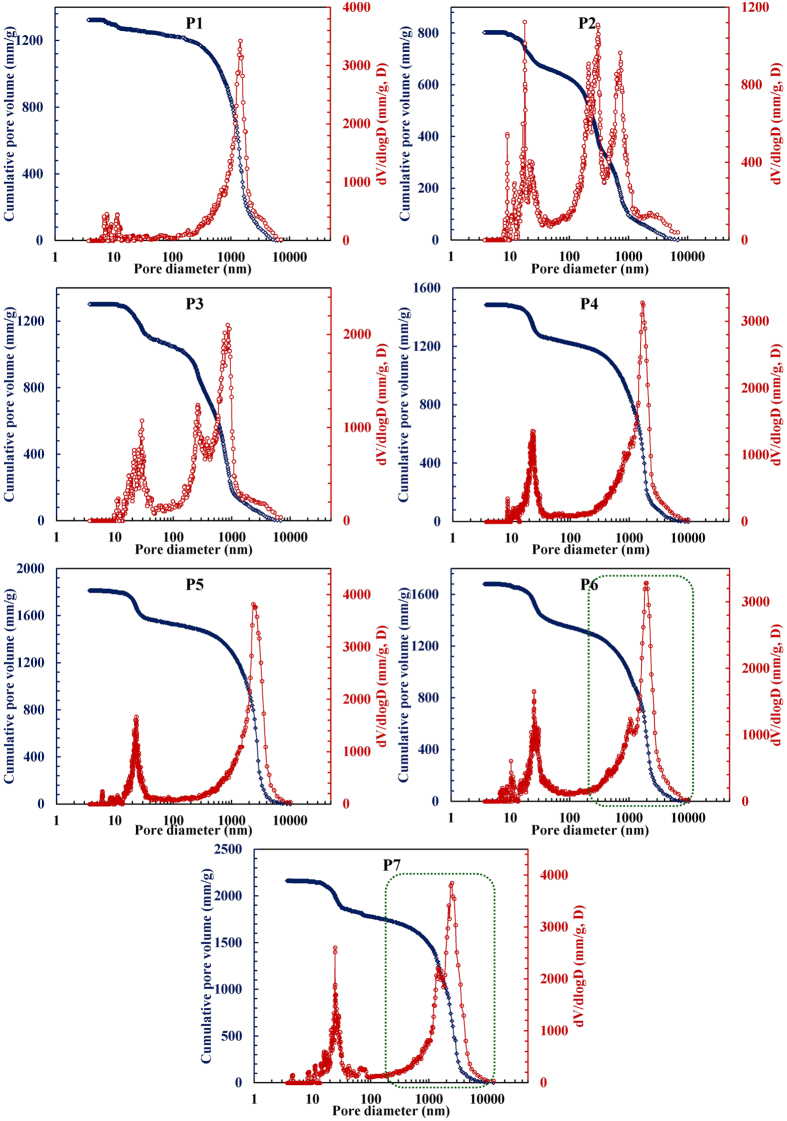
Pore size distributions of the GIS-NaP1 zeolite samples analysed by mercury intrusion porosimetry.

As higher water uptake is mainly caused by material’s macroporous character generated by the inter microsphere voids due to aggregation behaviour of the same in high Si/Al GIS-NaP1 zeolite samples, therefore, it becomes necessary to provide macropore distribution, bulk density, and other porosity data. Cumulative pore volume and differential pore volume curves corresponding to pore diameters for GIS-NaP1 zeolite samples analysed by mercury intrusion porosimetry are presented in Fig. 17. On a closely evaluation of the pore size distribution with increasing Si/Al ratio, we notice a clear change in the peak positioning and peak width. Cumulative pore volume as well as differential pore volume curves (Fig. 17) do not show any clear distinction among interstitial space and intra/inter microsphere void space for P2 and P3 samples. Contrary to that various pore accesses and existence of macroporous inter microsphere voids are easily detectable in the pore size distribution curves for other GIS-NaP1 zeolite samples especially P6 and P7 (Fig. 17). Broad bimodal (100–10000 nm) macroscopic pore size distribution curves for P6 and P7 samples (highlighted by doted green line in Fig. 17 P6 and P7) are consequence of aggregative behaviour of GIS-NaP1 microspheres. Furthermore, P2 sample has least porosity and highest bulk density (0.79 g cm^−1^) among all the GIS-NaP1 samples whereas reverse has been observed for P7 sample (supporting information SI6).

### Concluding remarks on physical and topological properties of GIS-NaP1 zeolite microspheres having different Si/Al ratio

The composition data indicate that the Si/Al ratio of the crystalline material is much lower than that of the initial precursor system (Table 1) and only a small difference in the Si/Al ratios of the final product was observed. So, that may be one of the reasons for not having regular trend in the variation of shape, size and morphology of GIS-NaP1 zeolite microspheres. Nevertheless, the initial system determines the ultimate chemical composition, and thus GIS-NaP1 zeolite samples with Si/Al ratios ranging from 5 to 11 have been synthesized. Although no regular trend in the topological properties was seen but it is evident that crystal growth and morphology of knobbed microspheres are clearly influenced by the both silicon and aluminium concentrations in the liquid phase of the crystallization. If we draw a growth trend of knobbed GIS-NaP1 microspheres after neglecting P1 sample then we found that the GIS-NaP1 zeolite crystals obtained from low Si/Al ratio reaction mixture have irregular and distorted morphology but with increase in Si/Al ratio, an improvement in morphology and enlargement of particles’ size was observed. Moreover, electron microscopic (Figs 2, 3, 4) and pore size distribution (Figs 13, 14 and 17) studies reveal that meso- and macro-porous character of zeolitic samples increase with increase in Si/Al ratio. Further, the meso- and macro-porous character originated form the self-assembling/aggregated primary crystallites, and secondary GIS-NaP1 zeolite microspheres (Fig. 5) plays a vital role in water vapour adsorption. Taking into account the particularities of zeolite crystallization system, and Davies and Jones^39^ model of crystal-growth and dissolution, Bosnar *et al.*^40^,^41^ defined the kinetics of zeolite crystal growth as:




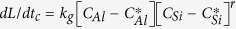




where *k*_g_ and *r* represent the growth rate constant and Si/Al ratio of crystallized zeolite, respectively. In this crystal growth equation C and C* are the concentration of respective element (reported in there subscript) in the liquid phase during crystallization and solubility of zeolite at given crystallization condition, respectively.

As per their findings growth rate is size independent but largely dependent on the reaction of monomeric and/or low-molecular aluminate, silicate and aluminosilicate anions in the liquid phase on growing crystal surface^40^. Moreover, increase in the ratio of Si/Al in liquid phase enhances the degree of polycondensation of silicate anions which leads to the morphology and phase transformation. As reported by Shirazi *et al.*^42^ in clear solution synthesis of ZSM-5 zeolite, increase in aluminium content i.e., decrease in Si/Al ratio, crystal size increases but contrary to that Araujo *et al.*^43^ reported that during ZSM-12 synthesis the average size of crystals decreases slightly as Si/Al ratio of materials increases.

## Discussion

The remarkably high water uptake capacity (1.39 g g^−1^) of as-synthesized highly phase pure GIS-NaP1 zeolite microspheres make them a potential applicant for different water adsorption-desorption based applications. High water uptake at medium vapour pressure (P/P_o_ = 0.1–0.3) and sharp increase in water loading at high relative pressure (P/P_o_ > 0.9) put forward them as a worthwhile adsorbent especially for TDCs and AHPs, because water loading at moderate/high vapour pressure reduces the useful loading lift within the possible cycle. In addition, the reported GIS-NaP1 zeolite material has nearly five times higher water adsorption capacity in comparison to the literature reported conventionally used zeolite as well as studied LTA and FAU zeolite. Moreover, an extensive comparison was made among the reported water adsorption capacities for common molecular sieves, aluminophosphates, titanosilicates, metal organic frameworks (MOFs), mesoporous materials, silica-alumina based materials etc. and as-synthesized GIS-NaP1 zeolite microspheres. Our materials show maximum adsorption potential (Table 5). The most exciting feature of the presented work is unusual multistep water vapour adsorption behaviour (i.e., *S*-shape or Type VI adsorption isotherm) of as-synthesized GIS-NaP1 which has never been reported before for any type of zeolite. This indicates that water uptake capacity of GIS-NaP1 zeolite is not just attributed to the available porosity but also to the hydrophilicity/hydrophobicity of material depending on Si/Al ratio, van der Waals forces of attractions, surface charge or polarity, possible structural transitions etc. As the extra-framework cations (charge balancing cations) of zeolite framework are mobile and exchangeable so these can be migrate to other adsorption sites during the water vapour adsorption depending upon their nature and position. Moreover, during the adsorption process water molecules also shows interaction with the cations and have tendency to make aqua-complex^5^,^44^,^45^. As there is a great scope to improve the surface functionality (by surfactant treatment), microporosity (using structure directing agents/templates, and various exchangeable cations) etc., so future work will be much focused on these aspects to further improve their adsorption potential and performance. Furthermore, micro-structural analysis reveals that Si/Al ratio has great influence on the shape, size and morphology of GIS-NaP1 zeolite microspheres, which consequentially affects the water adsorption capacity.

## Methods

### Materials preparation and characterisation

Single-phase GIS-NaP1 zeolite samples were synthesized by the traditional hydrothermal method using specially designed Teflon lined stainless autoclave as reported in previous work^1^. Phase pure GIS-NaP1 zeolite microspheres were synthesized from a gel having chemical composition Al_2_O_3_:xSiO_2_:14Na_2_O:840 H_2_O, where x is 10, 12, 14, 16, 18, 20 and 22. Based on the reaction mixture Si/Al ratio (5, 6, 7, 8, 9, 10 and 11), samples are coded as P1, P2, P3, P4, P5, P6 and P7, respectively. In each synthesis the reaction mixture was first stirred vigorously for 24 h in the closed autoclave at a stirring speed of 1800 rpm at 298 K to ensure the homogeneous mixing of hydro-gel and then crystallization was done by heating the aged gel at 373 K with 1000 rpm stirring speed at constant pressure. A solid product was recovered from aqueous solution by high speed centrifugation. For each sample at least four washings were carried out and the final product was dried at 333 K and stored in powder form for further use.Initial reactants used for these preparations were: sodium aluminate solution (Al_2_O_3_:Na_2_O = 1.1–1.3, Showa Chemical Co. Ltd., Japan), water glass (sodium silicate, SiO_2_:Na_2_O = 2.06–2.31, Wako Pure Chemical Ind. LTD., Japan), sodium hydroxide (min. 96%, Junsei Chemical Co. Ltd., Japan) and deionized water (DIW).

Powder XRD studies of GIS-NaP1 zeolite samples were made on PANalytical: X’Pert PRO diffractometer with Cu-Kα radiation (λ = 1.5418 Å) and the data was collected in 2θ range 10–50° with a step size of 0.02° s^−1^. Phase identification was performed with the help of JCPDS file (PDF#97-000-9550) for inorganic compounds. In addition, surface morphological studies of GIS-NaP1 zeolite particles were also made with scanning electron microscope (SEM, JEOL-JSM-7000F). During the surface morphological studies of GIS-NaP1 zeolite particles on scanning electron micrograph, the elemental composition was measured with energy dispersive X-ray spectrometry (EDS). Transmission electron microscopic (TEM) patterns were obtained on a FEI Tecnai^TM^ G^2^ F30 electron microscope operating at 300 kV. Particle size and particle size distributions (PSD) were measured by light scattering analysis (Nanotrac Wave, Microtrac, Inc.) at 298 K. Additional information concerning the GIS-NaP1 zeolite framework formation during the growth process was obtained by further analysing the solid specimens using Fourier transformation infrared (FT-IR) spectroscopy. FT-IR spectroscopy was performed on a Jasco FT/IR 610 spectrometer using KBr wafer technique. A spectrum was collected in the mid-IR range from 400 to 4000 cm^−1^ with a resolution of 1 cm^−1^. The FTIR spectrum of GIS-NaP1 zeolite was taken to complete the information of long range order (XRD) with short range order (FTIR). Water vapour adsorption-desorption studies were carried out at 298 K using an automatic adsorption measurement apparatus, BELSORP-max (BEL Japan, Inc.). Before an adsorption measurement, each sample was outgassed for 6 h at 523 K. Brunauer-Emmet-Teller (BET) surface areas have been calculated from adsorption branch in the relative pressure range from 0.002 to 0.50. The water uptake in weight percentage (wt.%) term is calculated as [(Amount of water vapour adsorbed/Amount of adsorbent) × 100]. More than 65 point water vapour adsorption-desorption isotherm has been measured and used for calculation of total pore volume. The total pore volume was calculated by measuring the amount of adsorbed water vapour at 0.984P/P_o_. The micropore size distribution in the GIS-NaP1 zeolite samples have been calculated using Horvath-Kawazoe (HK) and Saito-Foley (SF) analysis method. Furthermore, for comparative water vapour adsorption studies five different high alumina commercially available zeolite molecular sieves and one self-synthesized NaA zeolite (LTA_SS_) have been used. The commercial zeolites used in this study are molecular sieves 3A (zeolite LTA_3A_), molecular sieves 4A (zeolite LTA_4A_), molecular sieves 5A (LTA_5A_), molecular sieves 13X (zeolite NaX) and molecular sieves NaY (zeolite NaY) and all were obtained from Sigma-Aldrich, USA. Further, to analyse the pore structure of GIS-NaP1 zeolite samples N_2_ adsorption–desorption measurements were performed at 77 K and Barrett-Joyner-Halenda (BJH) and Dollimore-Heal (DH) analyses were carried out to measure the mesoporosity of material. Mesopore volume (pore size 2–50 nm) of all GIS-NaP1 zeolite samples were calculated using BJH method. As t-plot does not fit well to obtain N_2_ adsorption data, therefore, the micropore volume has been calculated by subtraction from the total pore volume (BET_Total_) derived by the amount of N_2_ adsorbed at P/P_o_ = 0.99 to the mesopore volume (BJH_meso_) calculated by the BJH method. To describe the pore size distribution of the GIS-NaP1 zeolite samples in the full pore size range the non-local density functional theory (NLDFT) has been applied to the respective N_2_ adsorption-desorption isotherm. NLDFT involves complex mathematical modelling of fluid–solid interactions along with geometrical considerations (pore geometry) and thus, it provides an accurate structure of fluid confined in the pores. Apart from these adsorption studies, the water vapour and N_2_ adsorption data were also fitted to the Langmuir isotherm to calculate the Langmuir surface area. The thermal stability and thermal behaviour of GIS-NaP1 zeolite samples were ascertained by thermogravimetric analysis (TGA), derivative thermogravimetry (DTG) and differential thermal analysis (DTA). TGA-DTG-DTA data were recorded simultaneously on a TGA/DSC 1 STAR^e^ system METTLER TOLEDO instruments with an alumina crucible and alumina powder reference. Samples were heated up to a temperature of 1273 K in the presence of nitrogen atmosphere with a heating rate of 10 K min^−1^. The thermogravimetric analysis data has also been used to calculate hydrophilicity/hydrophobicity and water adsorption capacity of as-synthesized GIS-NaP1 zeolite samples as well as commercial zeolite materials. For all these thermos-gravimetric analysis, each dried sample was just kept in glass bottle at ATP and never been exposed to any controlled humid conditions. The values of hydrophobicity index and water adsorption capacity were calculated by using following equation^5^:




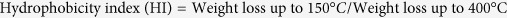







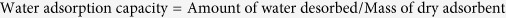




The zeta potential of dispersed GIS-NaP1 zeolite microspheres was determined in distilled water using zeta potential analyser (Zeta Finder, Matec Applied Sciences) at 25 °C (supplementary information SI5). Sample microspheres were added to the solute in a weight percentage of 0.5%. Intrusion/extrusion mercury measurements were performed using a PASCAL 440 (Thermos Scientific) mercury porosimetry with the following parameters: contact angle 140°, mercury surface tension 0.48 N m^−1^, and maximum intrusion pressure 400 MPa.

## Additional Information

**How to cite this article**: Sharma, P. *et al.* GIS-NaP1 zeolite microspheres as potential water adsorption material: Influence of initial silica concentration on adsorptive and physical/topological properties. *Sci. Rep.* **6**, 22734; doi: 10.1038/srep22734 (2016).

## Supplementary Material

Supplementary Information

## Figures and Tables

**Table 1:** Selected area electron dispersive spectroscopic (EDS) results for each as-synthesized GIS-NaP1 zeolite samples.

Sample	Si:Al^a^	Element	Weight%	Atomic%	Si:Al^b^
P1	5	O K	52.53	64.60	1.65
		Na K	11.38	9.74	
		Al K	13.26	9.67	
		Si K	22.83	15.99	
P2	6	O K	56.63	68.28	1.78
		Na K	10.58	8.88	
		Al K	11.49	8.22	
		Si K	21.30	14.63	
P3	7	O K	52.25	64.46	1.82
		Na K	10.36	8.89	
		Al K	12.91	9.45	
		Si K	24.48	17.20	
P4	8	O K	52.90	64.97	1.95
		Na K	11.22	9.59	
		Al K	11.82	8.61	
		Si K	24.06	16.83	
P5	9	O K	51.64	63.90	1.97
		Na K	10.66	9.18	
		Al K	12.36	9.07	
		Si K	25.33	17.85	
P6	10	O K	44.30	57.24	2.34
		Na K	8.22	7.40	
		Al K	13.83	10.59	
		Si K	33.65	24.77	
P7	11	O K	55.12	67.03	2.11
		Na K	10.32	8.73	
		Al K	10.80	7.79	
		Si K	23.75	16.45	

^a^Si/Al ratio of reaction mixture.

^b^Si/Al ratio of dried zeolite sample.

**Table 2:** Physical and water vapour adsorption properties of studied zeolite materials.

Material	BET Surface area, *m*^*2*^*g*^−*1*^	Pore volume, *cm*^3^*g*^−*1*^	Water vapour adsorption, *cm*^3^*g*^*−1*^		
			P/P_o_ = 0.1	P/P_o_ = 0.3	P/P_o_ = 0.98
LTA_3A_	819.4	0.25	261.1	280.2	306.6
LTA_4A_	839.0	0.25	272.0	286.0	314.2
LTA_5A_	818.8	0.26	265.2	285.6	315.8
LTA_SS_	880.4	0.26	281.0	298.5	318.6
NaX	1050.3	0.32	344.0	369.6	399.0
NaY	1259.1	0.33	351.4	376.6	409.0
P1	853.6	0.34	99.7	251.8	423.4
P2	818.3	0.32	98.7	240.0	527.9
P3	747.3	0.43	110.3	219.4	528.8
P4	719.5	0.97	82.6	220.6	1204.9
P5	654.1	0.64	119.1	216.2	799.2
P6	689.9	1.30	109.4	220.9	1616.2
P7	683.5	1.39	96.5	225.2	1727.5

**Table 3:** Textural parameters of as-synthesized GIS-NaP1 zeolite samples determined by N_2_ adsorption-desorption isotherms.

Materials	Surface area, *m*^*2*^*g*^−*1*^			Pore volume,*cm*^3^*g*^−*1*^			Micropore diameter, *nm*		Mesopore diameter, *nm*		
	BET	Langmuir	BJH	BET_Total_	BJH_Meso_	Micro_BET-BJH_	HK	SF	BET	BJH	DH
P1	29.19	21.36	24.56	0.11	0. 08	0.03	0.46	0.51	15.24	22.22	18.61
P2	41.37	29.45	34.51	0.20	0.14	0.06	0.46	0.51	19.63	39.35	33.06
P3	36.93	29.35	28.56	0.21	0.14	0.07	0.46	0.51	22.88	32.08	44.92
P4	42.66	33.52	35.94	0.24	0.17	0.07	0.46	0.51	22.40	40.52	38.48
P5	45.11	32.39	37.34	0.25	0.18	0.07	0.46	0.51	22.56	42.86	44.92
P6	43.48	33.79	32.33	0.29	0.16	0.13	0.46	0.51	26.68	37.64	44.92
P7	46.50	36.22	38.44	0.26	0.18	0.08	0.46	0.51	22.20	45.11	44.92

**Table 4:** Hydrophobicity index (HI) and water adsorption capacity values calculation for as-synthesized GIS-NaP1 zeolite samples based on the amount of water lost during thermo-gravimetric analysis.

Material	HI	Capacity,*mg g*^−*1*^	Material	HI	Capacity,*mg g*^−*1*^
LTA_3A_	0.679	221.31	P1	0.748	199.32
LTA_4A_	0.711	229.72	P2	0.755	200.82
LTA_5A_	0.606	241.05	P3	0.754	200.28
LTA_SS_	0.707	241.88	P4	0.749	197.40
NaX	0.677	313.52	P5	0.750	198.48
NaY	0.820	318.81	P6	0.748	191.12
—	—	—	P7	0.753	192.35

**Table 5:** Water adsorption capacities of reported micro-mesoporous materials, and studied commercial as well as synthesized zeolite samples.

Material	Capacity, *g g*^−*1*^	Material	Capacity, *g g*^−*1*^	Material	Capacity, *g g*^−*1*^	Material	Capacity,*g g*^−*1*^
NaX^44^	0.33	ETS-10^5^	0.14	AlPO-18^2^	0.30	SG^46^,^47^	0.06
KX^44^	0.26	HETS-10^5^	0.18	Cu-BTC^2^	0.32	SG/LiBr-17%^46^,^47^	0.22
RbX^44^	0.20	ALPO-5^5^	0.30	NaX^3^	0.19	SG/MgCl_2_-17%^46^,^47^	0.20
CsX(5)^44^	0.27	MCM-48^5^	0.83	MgNaX^3^	0.21	SG/CaCl_2_-17%^46^,^47^	0.33
CsX(30)^44^	0.22	KIT-1^5^	0.81	LiX^3^	0.24	SG/CaCl_2_-26%^46^,^47^	0.31
CsX(45)^44^	0.19	SBA-1^5^	0.44	CaNaA^3^	0.16	SG/CaCl_2_-33%^46^,^47^	0.28
Li-LSX^44^	0.35	SBA-15^5^	0.84	SG^3^	0.18	SG/Na_2_SO_4_^47^,^48^	0.85
Na-LSX^44^	0.30	SiMCM-41^5^	0.71	AS/CaCl_2_^3^	0.18	SBA-15/CaCl_2_-43%^49^	0.47
K-LAX^44^	0.25	AlMCM-41^5^	0.84	Silica aerogel^50^	1.35	MCM-41/CaCl_2_-42%^49^	0.51
CaY^5^	0.34	AlMCM-41^5^	0.77	Alumina aerogel^50^	1.25	SG/CaCl_2_-33.7%^49^	0.42
HY^5^	0.20	FSM-16^5^	0.78	Mixed SiO_2_–Al_2_O_3_^50^	1.15	Bentonite/CaCl_2_^47^,^51^	0.45
KY^5^	0.29	MIL-101(Cr)^6^,^17^	1.01	SiO_2_ aerogel/LiBr^52^	0.80	Kaolinite/CaCl_2_^47^,^51^	0.30
LiY^5^	0.36	MIL-101(Cr)^6^,^17^	1.43	SiO_2_ aerogel/CaCl_2_^53^	1.17	Attapulgite (AT)^54^	0.11
RbY^5^	0.34	MIL-101(Cr)^6^,^17^	1.37	SiO_2_ xerogel/CaCl_2_^53^	1.13	AT/LiCl-10%^54^	0.20
CaNaA^5^	0.16	MIL-100(Fe)^6^,^17^	0.65	SG type A^55^	0.40	AT/LiCl-20%^54^	0.26
NaA^56^	0.29	MIL-100(Cr)-Cl^6^,^17^	0.67	SG type RD^55^	0.45	AT/LiCl-30%^54^	0.44
MgA^56^	0.42	MIL-100(Cr)-F^6^,^17^	0.61	SG/CaCl_2_^57^	0.80	AT/LiCl-40%^54^	0.44
NaY^56^	0.34	MIL-100(Cr)-SO_4_^6^,^17^	0.61	SG/CaCl_2_^58^	0.60	13X zeolite^54^	0.20
MgY^56^	0.42	HKUST-1^6^,^17^	0.42	Zn(NDI-H)^59^	0.44	SG^54^	0.25
NaX^56^	0.34	HKUST-1^6^,^17^	0.55	Zn(NDI-NHEt)^59^	0.30	Vermiculite/LiNO_3_^60^	0.51
MgX^56^	0.45	ISE-1^6^,^17^	0.22	Zn(NDI-SOEt)^59^	0.29	Kaolin/CaCl_2_^47^,^61^	0.30
AlPO-36, 37, 40^5^	0.30–0.33	Basolite™ C300^6^,^17^	0.6	MIL-101-H^63^	1.29	Wakkanai siliceous shale 62	0.27
AlPO-41, SAPO-41^5^	0.22–0.28	Basolite™ A100^6^,^17^	0.2	MIL-101-NH_2_^63^	0.81	WSS/LiCl-5%^62^	0.39
AlPO-8, 34, 35, 42, 43, 44, 47^5^	0.30–0.35	Basolite™ F300^6^,^17^	0.3	MIL-101-NH_2_:-UR2^63^	0.55	WSS/LiCl-10%^62^	0.58
AlPO-14, 17, 26, 33, 39^5^	0.23–0.28	MOF-801-P^18^	0.36	MIL-101-NH_2_:-Mal^63^	0.71	WSS/CaCl_2_-5%^62^	0.32
AlPO-20, 25, 28^5^	0.17–0.21	MOF-801-SC^18^	0.28	MIL-101-NH_2_:-3SO_3_H^63^	0.70	WSS/NaCl-5%^62^	0.35
LiNaX^64^	0.38	MOF-802^18^	0.09	MIL-101^a^-H:COOH^63^	1.02	WSS/NaCl-10%^62^	0.49
SAPO-34^64^	0.28	UiO-66^18^	0.43	MIL-101^b^-H:COOH^63^	0.91	AC1/Na_2_SiO_3_-10%^47^,^65^	0.17
AlPO-5^64^	0.24	MOF-808^18^	0.59	AC^11^,^47^	0.67	AC2/Na_2_SiO_3_-10%^47^,^65^	0.23
AlPO-17^64^	0.28	MOF-841^18^	0.51	OAC^11^,^47^	0.56	AC07/Na_2_SiO_3_/CaCl_2_^47^,^66^	0.85
AlPO-18^64^	0.39	DUT-67^18^	0.50	RAC (N_2_)^11^,^47^	0.60	AC12/Na_2_SiO_3_/CaCl_2_^47^,^66^	0.50
Attagulgite/CaCl_2_^64^	0.40	PIZOF-2^18^	0.68	RAC (H_2_)^11^,^47^	0.62	A1-6.7^47^,^67^	0.55
NaEMT^5^	0.32	MOF-804^18^	0.23	ROAC (N_2_)^11^,^47^	0.73	A2-7.7^47^,^67^	0.53
CaL^5^	0.20	MOF-805^18^	0.33	ROAC (H_2_)^11^,^47^	0.76	A3-8.7^47^,^67^	0.46
KL^5^	0.17	MOF-806^18^	0.34	SI 144 B^68^	0.94	Zeolite 3A^47^,^67^	0.21
ZSM20^5^	0.46	Mg-MOF-74^18^	0.60	HMS 3^68^	0.76	SG^47^,^67^	0.35
ZSM34^5^	0.29	Co-MOF-74^18^	0.50	AL 300^68^	0.58	FAM-Z05^69^	0.21
Na-Omega^5^	0.27	Ni-MOF-74^18^	0.49	TI 68	0.16	Carbon aerogel^70^	0.13
Mordenite^5^	0.15	CAU-6^18^	0.30	ZR 3^68^	0.10	a-Carbon aerogel-41^70^	0.38
Erionite^5^	0.22	CAU-10^18^	0.29	NB 3^68^	0.09	a-Carbon aerogel-54^70^	0.54
Analcime^5^	0.05	Basolite A100^18^	0.20	Z25^71^	0.14	HKUST-1^47^,^72^	0.55
KP^5^	0.12	Basolite A300^18^	0.65	Z40^71^	0.14	MIL-100(Fe)^47^,^72^	0.81
NaP^5^	0.24	Basolite C300^18^	0.55	Z40-H^71^	0.45	MIL-101(Cr)^47^,^72^	1.28
CaP^5^	0.22	Zeolite 13 × 18	0.33	Z40-HW^71^	0.47	DUT-4^47^,^72^	0.28
VPI^5^	0.35	MCM-41^18^	0.77	1-MOF^73^	0.20	ZIF^47^,^72^	0.02
SAPO^5^	0.55	BPL carbon^18^	0.00	2-MOF^73^	0.29	LTA_3A_^This work^	0.25
VAPO^5^	0.14	Clinoptilolite-K^75^	0.06	SG^47^,^74^	0.09	LTA_4A_^This work^	0.25
GeAPO-5, MnAPO-5^5^	0.32–0.36	Clinoptilolite-Na^75^	0.10	SG/Li10^47^,^74^	0.47	LTA_5A_^This work^	0.25
SAPO-5^5^	0.46	Clinoptilolite-Ca^75^	0.13	SG/Li20^47^,^74^	0.72	LTA_SS_^This work^	0.26
SAPO-40^5^	0.46	SG^2^	0.11	SG/Li30^47^,^74^	0.90	NaX^This work^	0.32
AlPO-11, MgAPO-11, SAPO-11^5^	0.12–0.18	NaA^2^	0.09	SG/Li40^47^,^74^	1.20	NaY^This work^	0.33
VAPO-11^5^	0.09	LiA^2^	0.10	SG^47^,^76^	0.23	P1^This work^	0.34
SAPO-42^5^	0.27	Li-LSX^2^	0.12	SG/LiCl^47^,^76^	0.67	P2^This work^	0.42
GaAPO-44^5^	0.20	PbY^2^	0.15	SG/Ca(NO_3_)_2_^47^,^77^	0.21	P3^This work^	0.43
GeAPO-34^5^	0.27	NiY^2^	0.15	SG/LiNO_3_^47^,^78^	0.22	P4^This work^	0.97
SAPO-17^5^	0.54	NaY^2^	0.15	SG-Meso-A/50CaCl_2_^79^	0.42	P5^This work^	0.64
SAPO-20^5^	0.34	LiY^2^	0.19	SG-Meso-B/50CaCl_2_^79^	0.47	P6^This work^	1.30
NaETS-4^5^	0.15	LaNaY^2^	0.19	SG-Micro/50CaCl_2_^79^	0.28	P7^This work^	1.39
CaETS-4^5^	0.19	SAPO-34^2^	0.28	SG/MgSO_4_^80^	0.40		

SG is short abbreviation for silica gel.
