# Supplementary material for: GIS-NaP1 zeolite microspheres as potential water adsorption material: Influence of initial silica concentration on adsorptive and physical/topological properties
Source: Sci Rep. 2016 Mar 11;6:22734. doi: 10.1038/srep22734 (PMC4786819; doi:10.1038/srep22734)
Supplement: Supplementary Information [file srep22734-s1.pdf]

Supplementary Information (SI)

GIS-NaP1 zeolite microspheres as potential water adsorption material: Influence of initial silica concentration on adsorptive and physical/topological properties

Pankaj Sharma, Ju-Sub Song, Moon Hee Han, Churl Hee Cho*

Graduate School of Energy Science and Technology, Chungnam National University, 99 Daehak-ro, Yuseong-gu, Daejeon 305-764, Republic of Korea

*Corresponding author. Tel.: +82 42 821 8606; fax: +82 42 821 8839.

E-mail address: choch@cnu.ac.kr (C.H. Cho), sharmapankaj47@yahoo.com (P. Sharma)

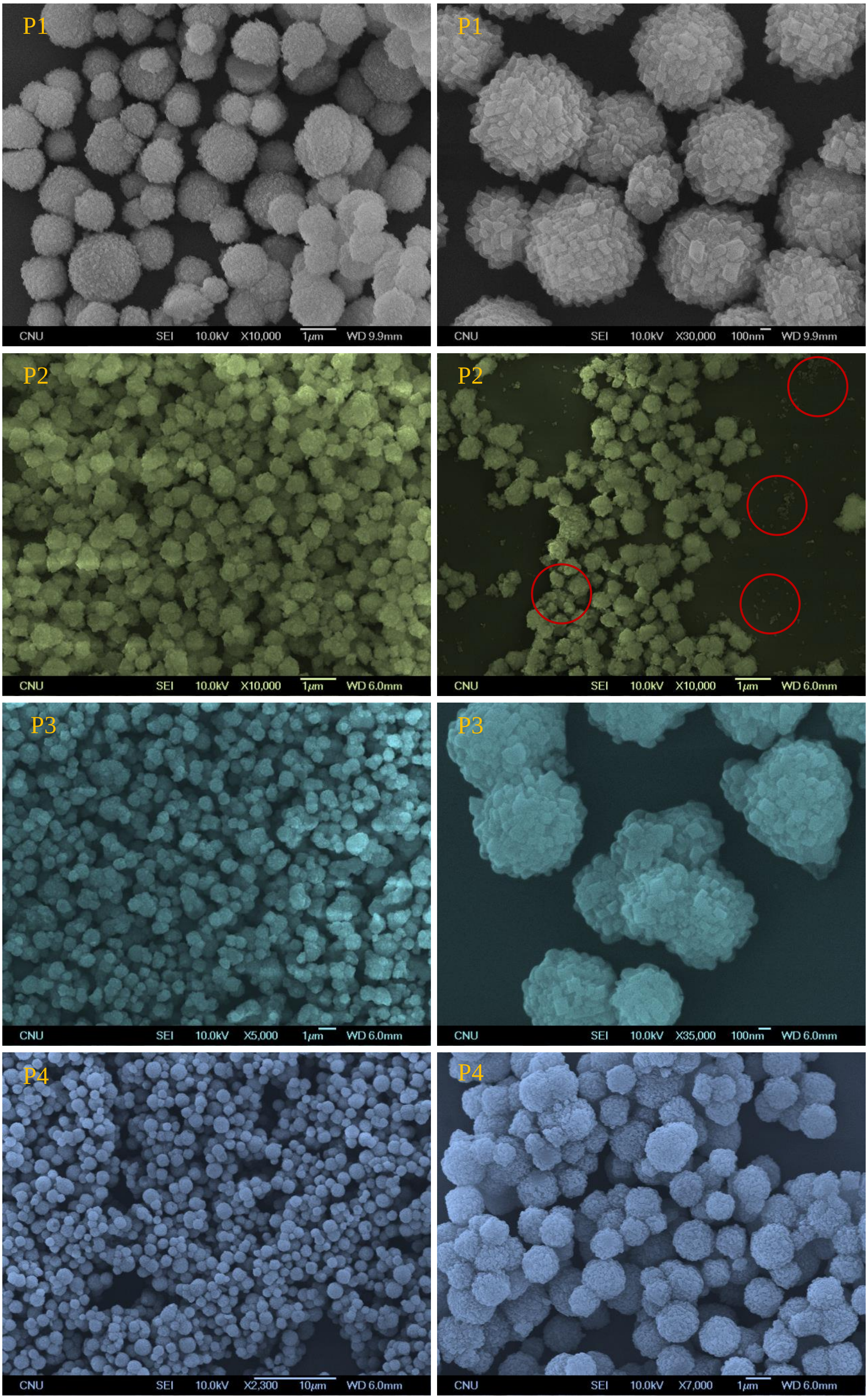

SI1 SEM image of GIS-NaP1 zeolite samples (continue).

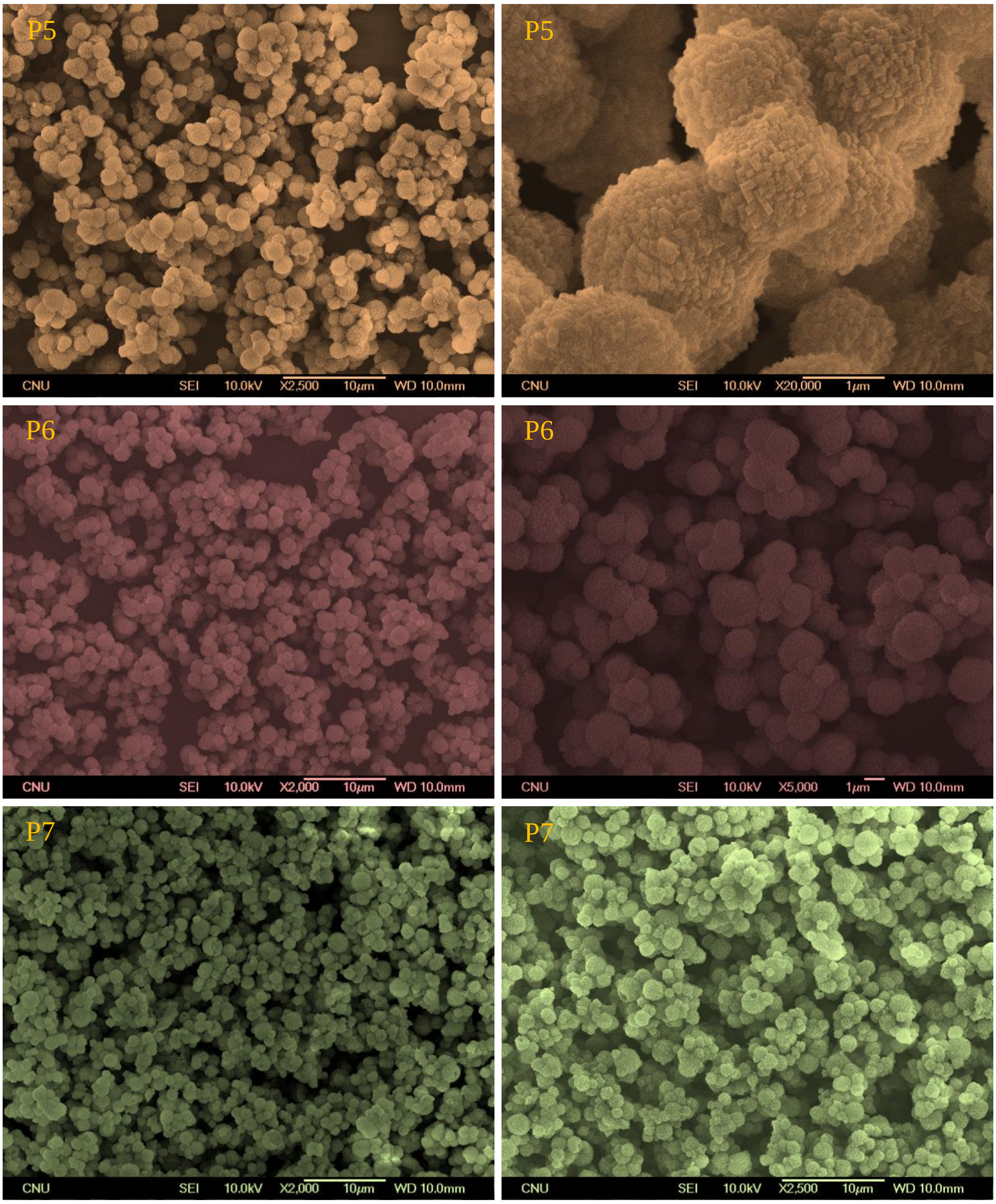

SI1 SEM image of GIS-NaP1 zeolite samples.

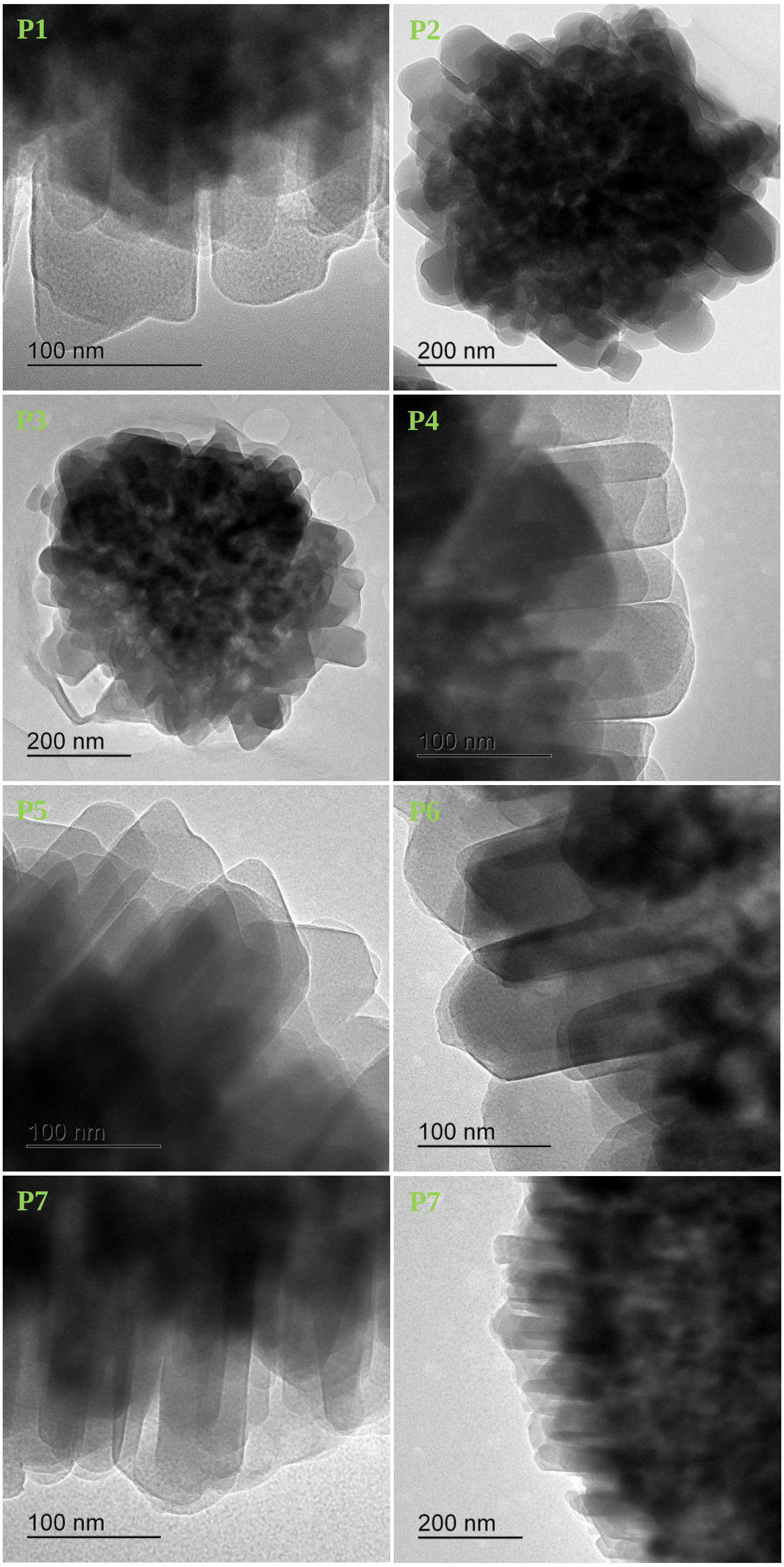

SI2 TEM images of GIS-NaP1 zeolite samples reveal the existence of inter crystallite voids or free space.

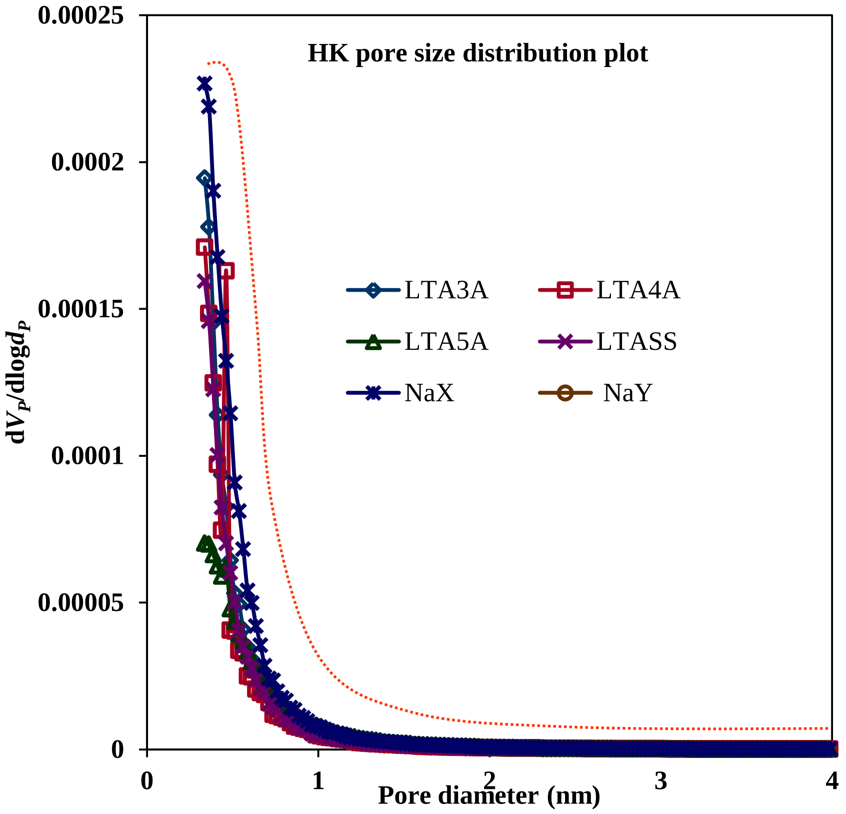

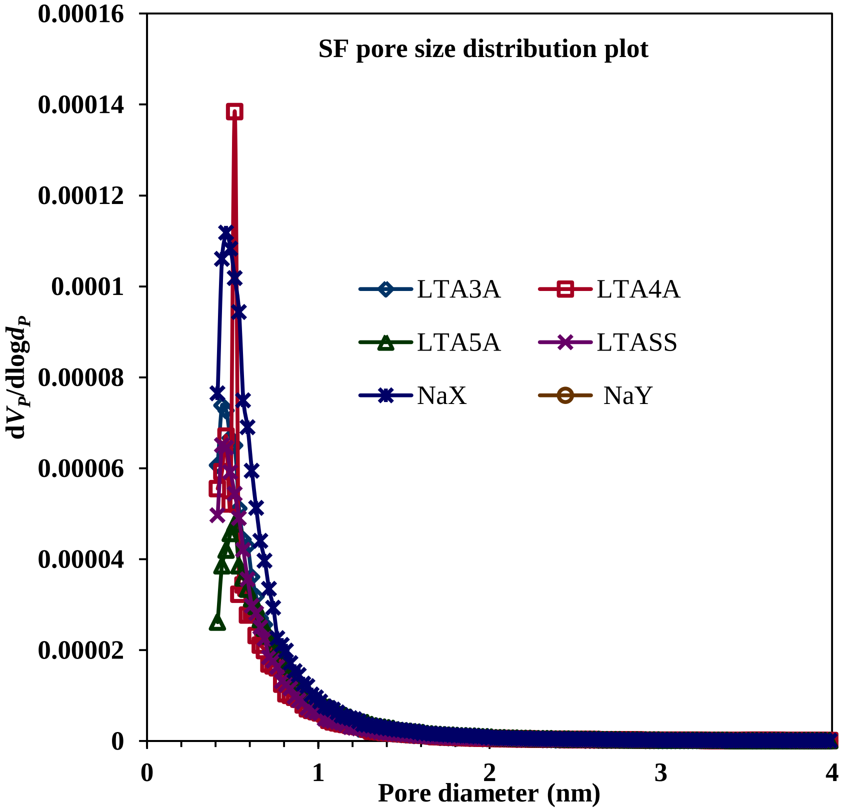

SI3 Water vapor adsorption HK and SF pore size distribution curves for commercial LTA (3A, 4A and 5A), NaX, NaY, and self-synthesized (SS) LTA zeolite crystals those used for comparative water vapor adsorption studies.

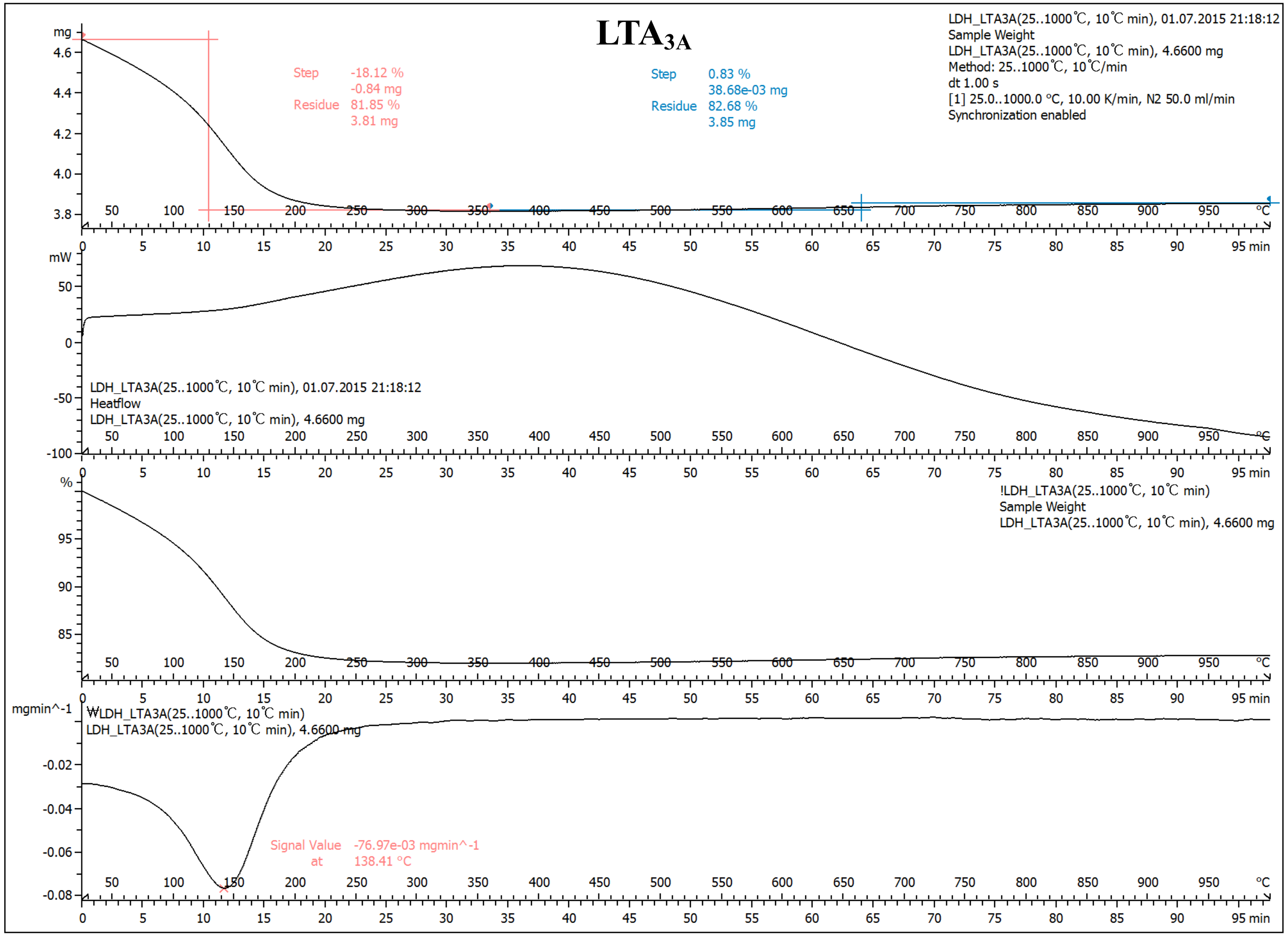

SI4 Thermo-gravimetric analysis (TGA), derivative thermogravimetry (DTG) and differential scanning calorimetry (DSC) curves of the commercial LTA (3A, 4A and 5A), NaX, NaY, and self-synthesized (SS) LTA zeolite crystals those used for comparative water vapor adsorption studies (continue).

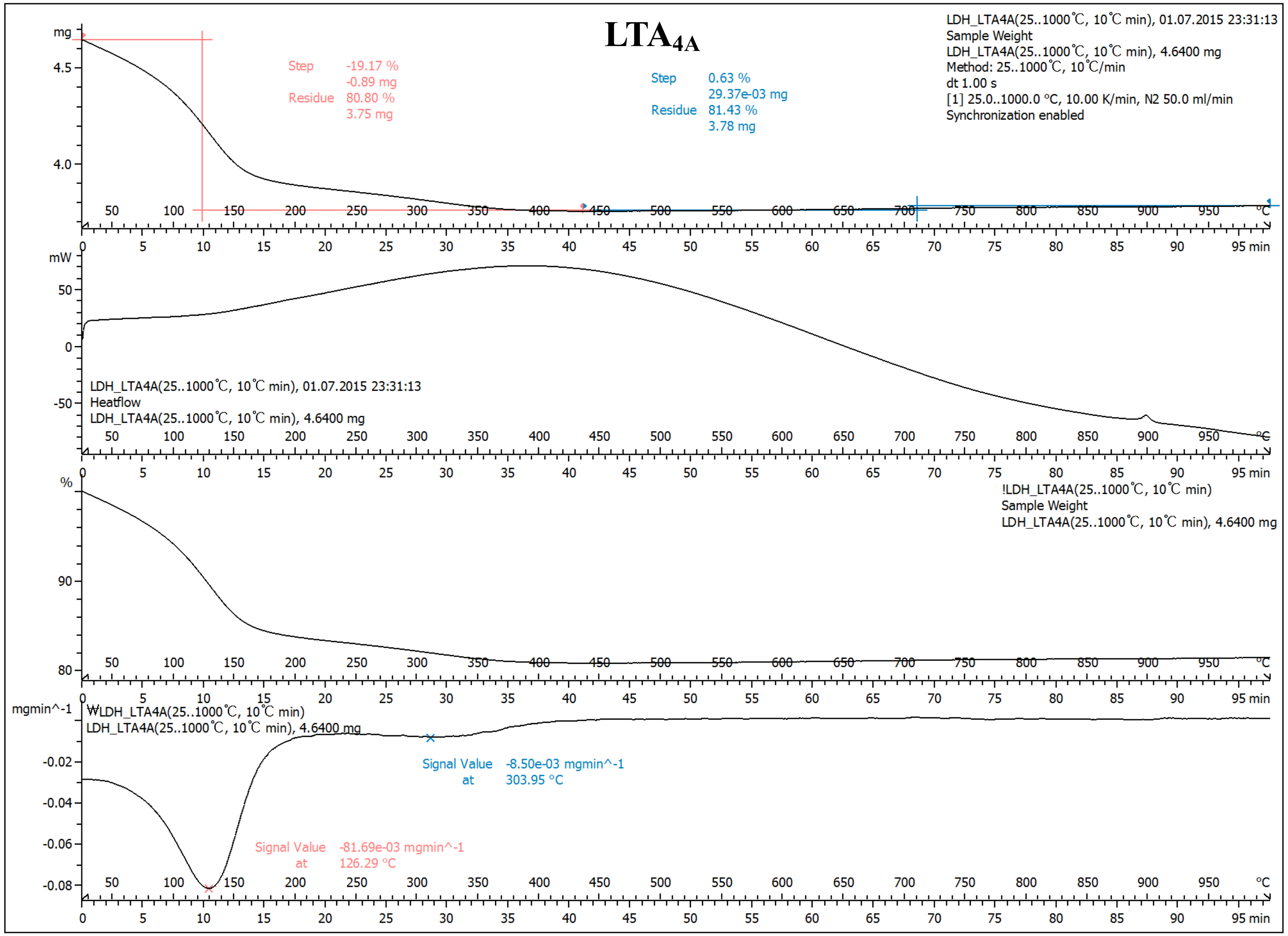

SI4 (continue)

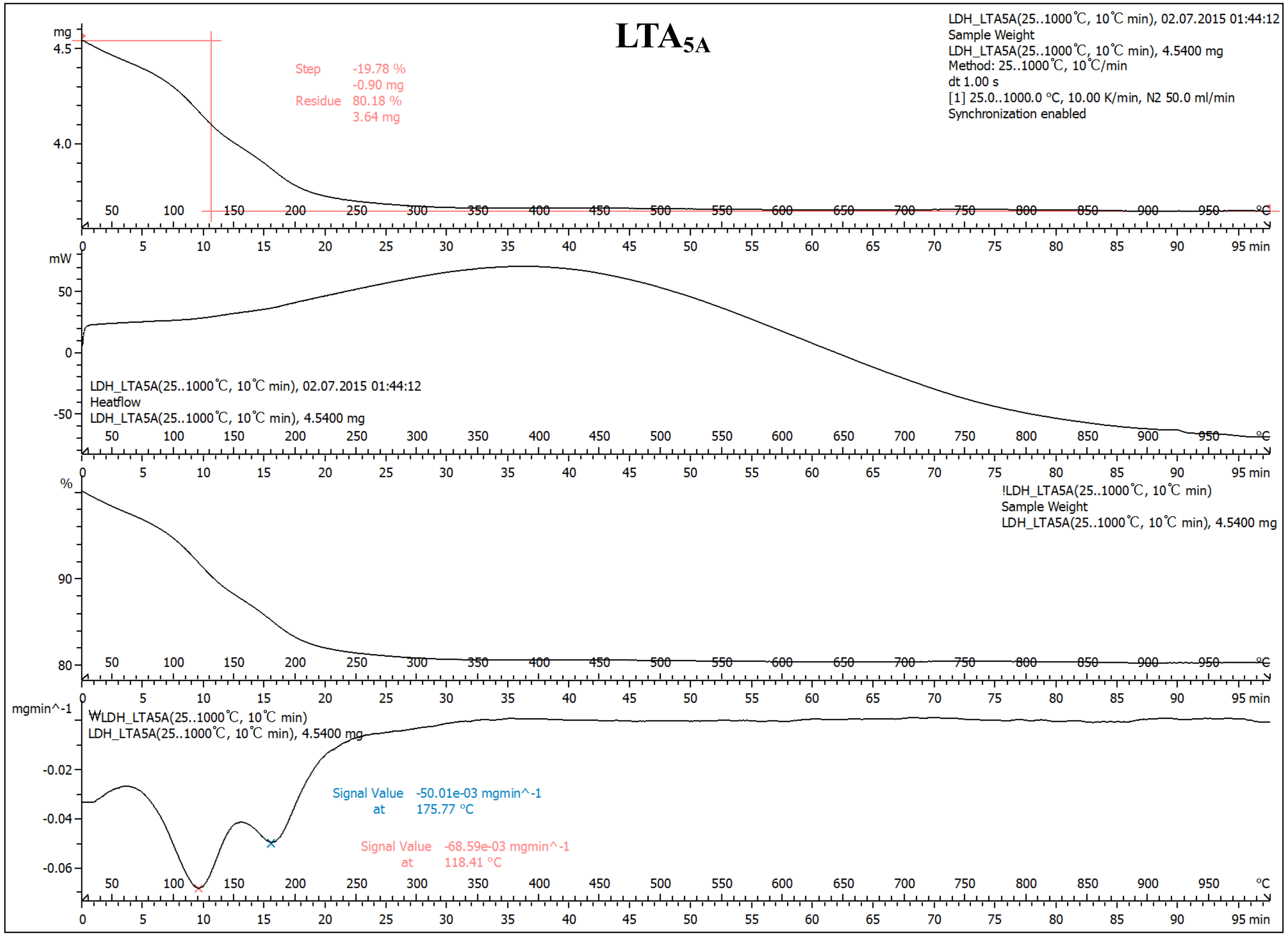

SI4 (continue)

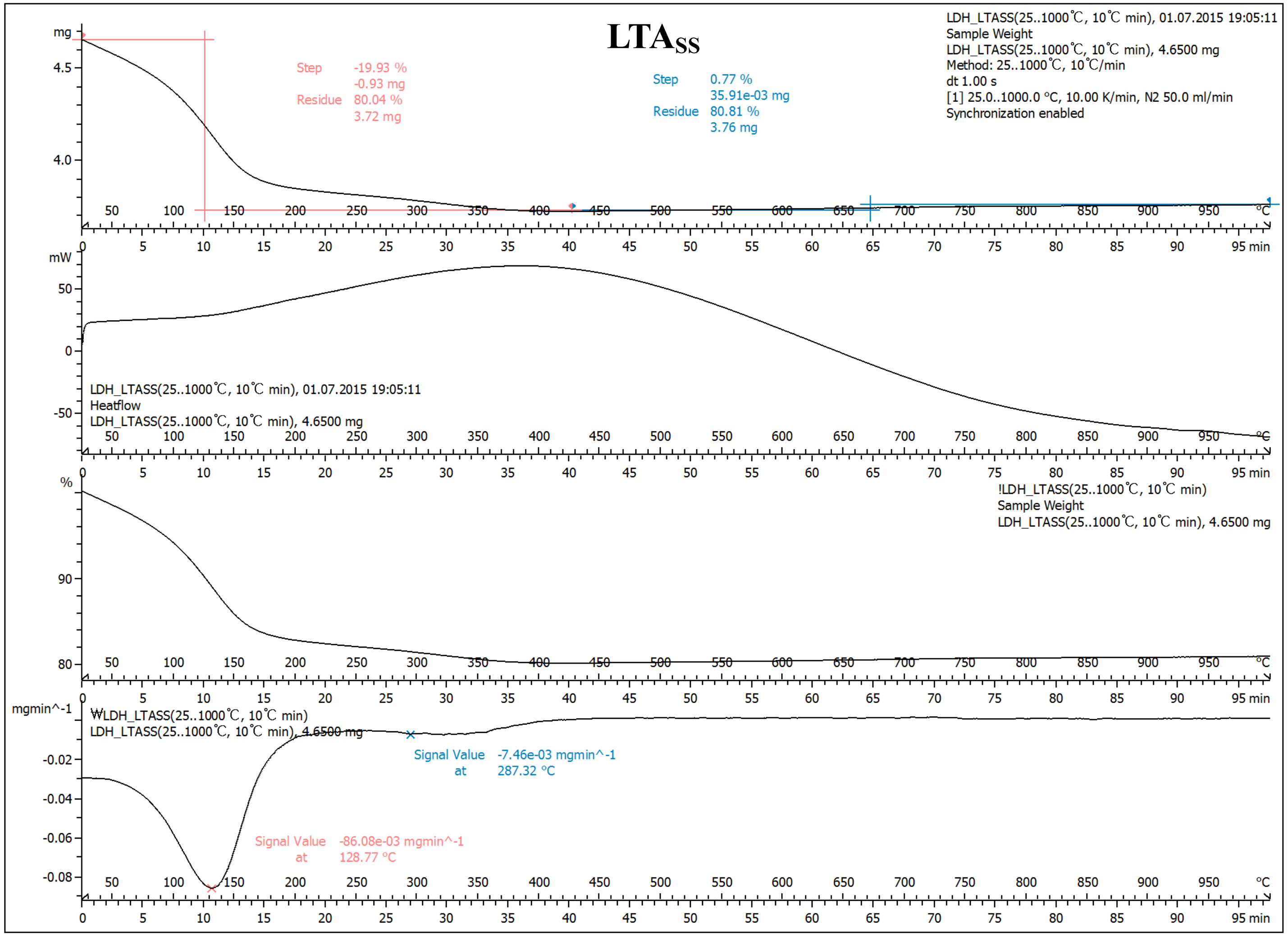

SI4 (continue)

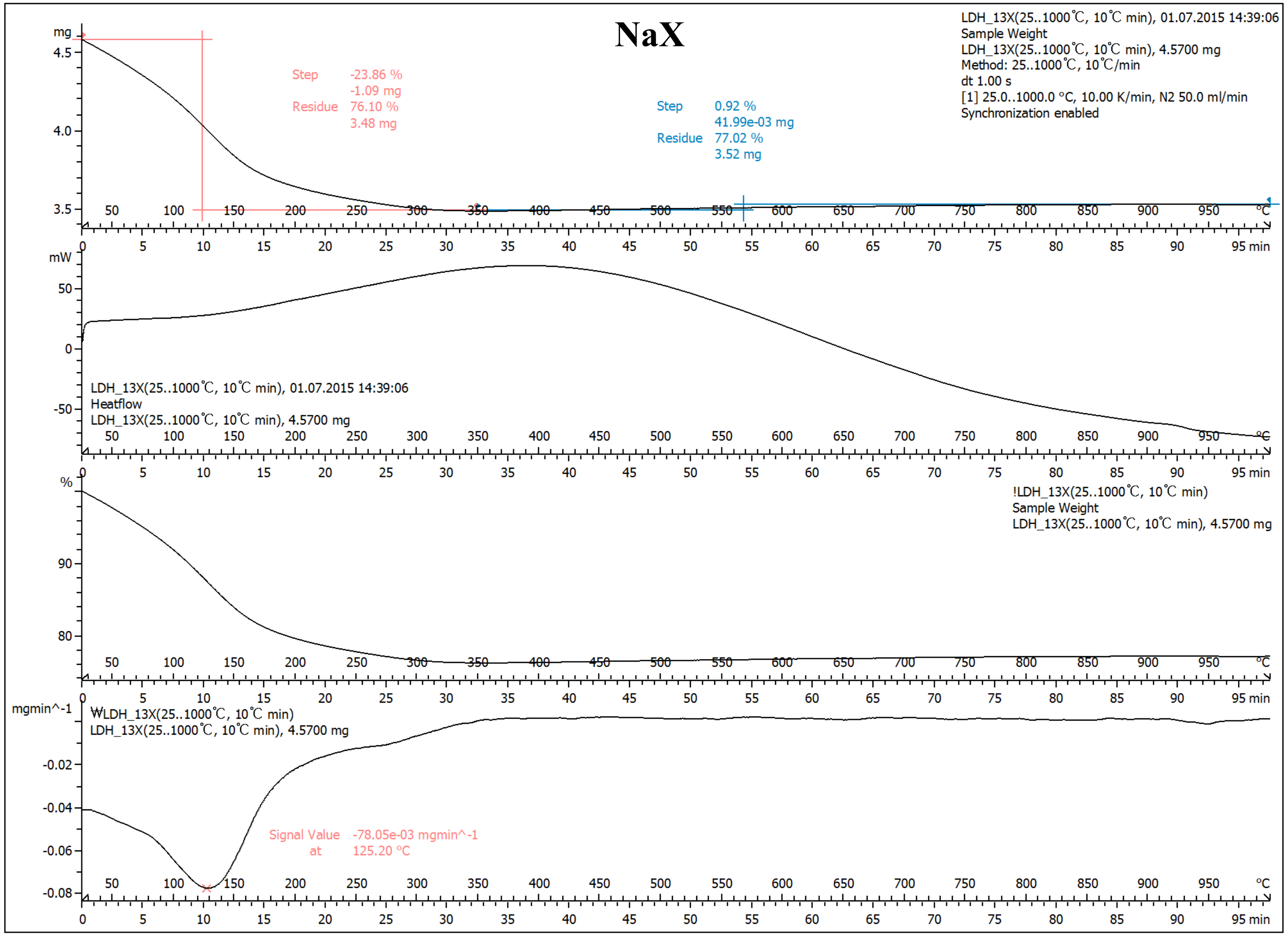

SI4 (continue)

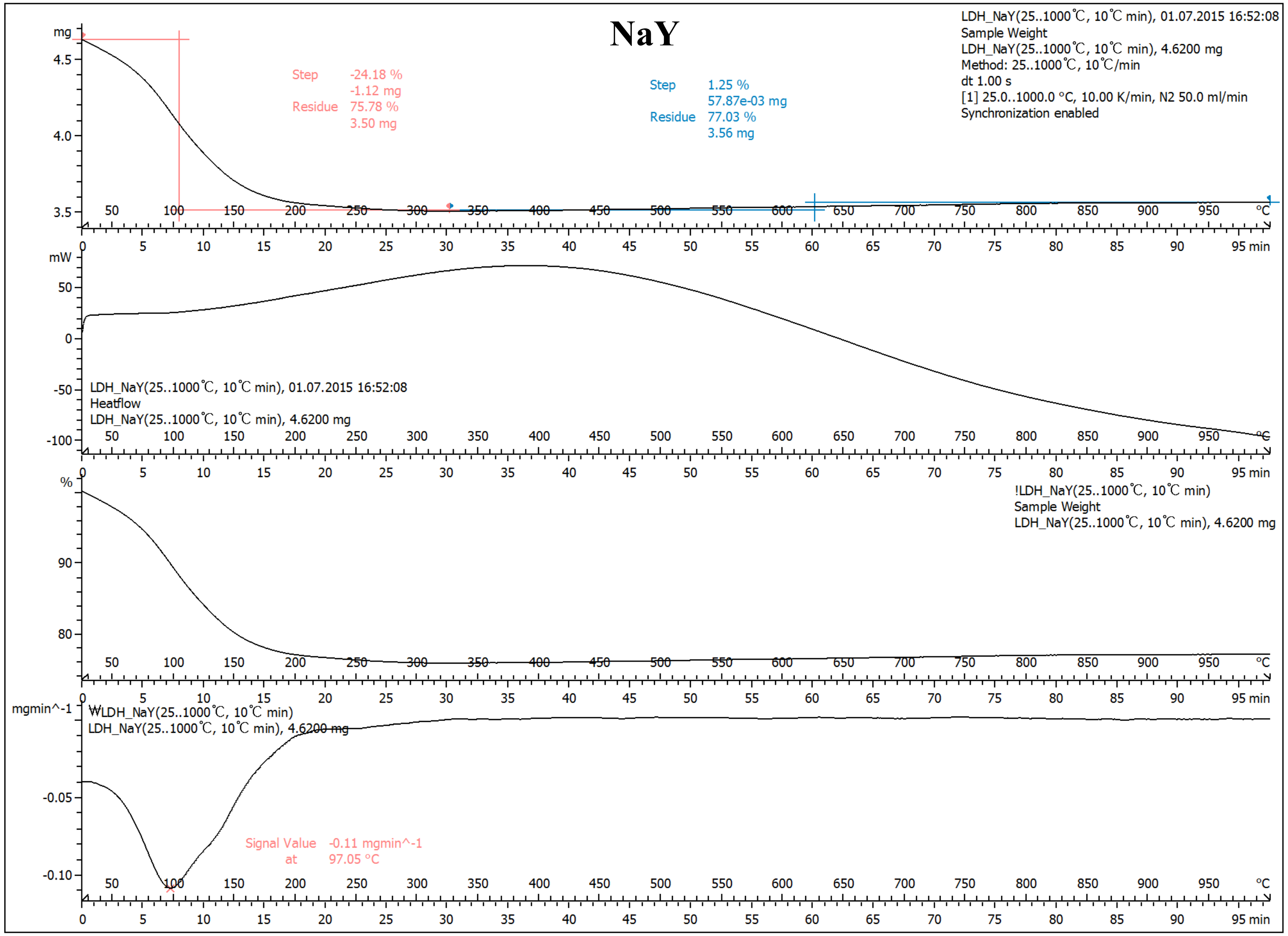

SI4 Thermo-gravimetric analysis (TGA), derivative thermogravimetry (DTG) and differential scanning calorimetry (DSC) curves of the commercial LTA (3A, 4A and 5A), NaX, NaY, and self-synthesized (SS) LTA zeolite crystals those used for comparative water vapor adsorption studies.

SI5 Single point zeta potential measurements of GIS-NaP1 zeolite microspheres dispersed in deionized water at 298 K

Materials	pH	Zeta potential (mV)
P1	9.45	-32.58
P2	8.66	-17.52
P3	9.61	-16.86
P4	9.84	-36.32
P5	8.90	-18.98
P6	8.88	-18.40
P7	9.70	-40.32

SI6 Mercury intrusion porosimetry data of GIS-NaP1 zeolite microspheres

Materials	Porosity by Hg intrusion (%)	Bulk density (g cm^{-3})	Total Intruded volume (cm^3 g^{-1})
P1	68.83	0.52	1.32
P2	63.69	0.79	0.80
P3	73.65	0.57	1.30
P4	74.12	0.50	1.48
P5	75.38	0.42	1.81
P6	78.30	0.46	1.68
P7	76.89	0.35	2.16
